# Human roars communicate upper-body strength more effectively than do screams or aggressive and distressed speech

**DOI:** 10.1371/journal.pone.0213034

**Published:** 2019-03-04

**Authors:** Jordan Raine, Katarzyna Pisanski, Rod Bond, Julia Simner, David Reby

**Affiliations:** 1 Mammal Vocal Communication and Cognition Research Group, University of Sussex, Brighton, United Kingdom; 2 Equipe Neuro-Ethologie Sensorielle, ENES/Neuro-PSI CNRS UMR 9197, Bioacoustics Team, University of Lyon/Saint-Etienne, Saint-Etienne, France; 3 *MULTISENSE* Research Lab, University of Sussex, Brighton, United Kingdom; University of Birmingham, UNITED KINGDOM

## Abstract

Despite widespread evidence that nonverbal components of human speech (e.g., voice pitch) communicate information about physical attributes of vocalizers and that listeners can judge traits such as strength and body size from speech, few studies have examined the communicative functions of human nonverbal vocalizations (such as roars, screams, grunts and laughs). Critically, no previous study has yet to examine the acoustic correlates of strength in nonverbal vocalisations, including roars, nor identified reliable vocal cues to strength in human speech. In addition to being less acoustically constrained than articulated speech, agonistic nonverbal vocalizations function primarily to express motivation and emotion, such as threat, and may therefore communicate strength and body size more effectively than speech. Here, we investigated acoustic cues to strength and size in roars compared to screams and speech sentences produced in both aggressive and distress contexts. Using playback experiments, we then tested whether listeners can reliably infer a vocalizer’s actual strength and height from roars, screams, and valenced speech equivalents, and which acoustic features predicted listeners’ judgments. While there were no consistent acoustic cues to strength in any vocal stimuli, listeners accurately judged inter-individual differences in strength, and did so most effectively from aggressive voice stimuli (roars and aggressive speech). In addition, listeners more accurately judged strength from roars than from aggressive speech. In contrast, listeners’ judgments of height were most accurate for speech stimuli. These results support the prediction that vocalizers maximize impressions of physical strength in aggressive compared to distress contexts, and that inter-individual variation in strength may only be honestly communicated in vocalizations that function to communicate threat, particularly roars. Thus, in continuity with nonhuman mammals, the acoustic structure of human aggressive roars may have been selected to communicate, and to some extent exaggerate, functional cues to physical formidability.

## Introduction

In competitive contests, evolutionary selection processes favour vocal communication of resource holding potential to settle disputes without engaging in potentially costly combat [1]. For example, many terrestrial mammalian species, including giant pandas [2], sea lions [3], fallow and red deer [4,5], and domestic dogs [6] use acoustic cues to body size or dominance rank in aggressive vocalizations to mediate agonistic interactions, particularly during male-male competition.

Among humans, the nonverbal components of speech also allow listeners to assess body size from the voice, including height and weight [7–10]. Yet, few studies provide evidence that human listeners can assess physical strength from the human voice. Sell et al. [11] found that actual strength explained 18% and 7% of the variance in listeners’ voice-based strength attributions of male and female vocalizers, respectively, when listeners were presented with short speech utterances. A more recent study showed that listeners were also able to judge the strength and height of unseen vocalizers relative to their own strength and height, from both aggressive speech utterances and aggressive roars [12]; however, that study did not examine the acoustic correlates of strength or body size nor whether these predicted listeners’ judgments. Indeed, despite the apparent capacity for listeners to gauge strength from the voice, the acoustic correlates of strength remain largely unknown following null or inconsistent results of past work [11,13–17].

Due to an evolutionary continuity in both structure and function between the vocalizations of other mammals and human nonverbal vocalizations, such as laughter [18–21] and infant distress screams [22–24], human nonverbal vocalizations may communicate evolutionarily and socially relevant information more effectively than speech, which is also relatively more constrained by linguistic content. Indeed, recent work has shown that human laughter (e.g., [25,21,26] but see [27]), tennis grunts [28], and simulated pain cries [29] all convey ecologically relevant cues to vocalizer traits that listeners utilize in their biosocial judgments. At the same time, while past studies show that listeners can estimate absolute strength from modal speech [11] and relative strength from both speech and roars [12], roars appear to exaggerate the expression of threat, as listeners judge male vocalizers as relatively stronger and larger than themselves when those vocalizers are producing roars compared to aggressive speech [12]. The information carried by nonverbal vocalizations may also be context-specific. For example, aggressive roars may communicate, or exaggerate, physical strength more effectively than fear screams.

To test these hypotheses, we compared the ability of listeners to estimate physical strength from human speech and from nonverbal vocalizations produced in two hypothetical contexts: aggression and distress. In these two distinct agonistic contexts, nonhuman mammals typically produce acoustically and perceptually distinct vocalizations that follow Morton’s motivational-structural rules [30]; hence, capitalising on perceptual associations between low frequency sounds and large size or dominance [31], aggressive vocalizations (roars, barks or growls) are typically structurally noisy and low-pitched [30–32]. In contrast, distress vocalizations are higher-pitched and usually (but not always) tonal, exploiting perceptual associations between high frequencies and small size or submission [30,31,33]. While aggressive vocalizations are thought to function to display threat and physical formidability, distress vocalizations typically function to solicit aid [34–36].

Like other mammals, humans produce roar-like vocalizations in aggressive contexts such as battle [37–39], and scream-like vocalizations in distress contexts [40]. Furthermore, women, who are on average physically weaker than men [41–43], are more likely to scream in response to threat scenarios than are men, whose responses are typically biased towards aggression [40].

Following the hypothesis that human roars and screams are homologous to mammalian vocalizations produced in aggressive and distress contexts, respectively, and are likewise affected by anatomical and physiological constraints, we may expect that the acoustic structure of these nonverbal vocalizations encodes honest information about the physical characteristics of the vocalizer [44–50]. However, we may also expect vocalizations produced in an aggressive context (hereafter roars) to function to maximize the expression of threat relative to those produced in a distress or submissive context (screams), which may in turn minimize perceived threat.

### The present study

In a recent paper we showed that listeners can judge the strength and height of others (relative to their own) from aggressive speech and roars, and that roars, while communicating honest information about strength and body size, also exaggerate these physical traits compared to aggressive speech among men [12]. While those results support the prediction that roars function to maximize the expression of formidability and threat, the study lacked acoustic data to examine the vocal correlates of strength and body size in nonverbal vocalisations and speech, or to link these acoustic parameters to listeners’ judgments of strength and size, and contained no data on screams or distressed speech.

Here, we thus build on previous research by comparing the acoustic structure of roars, screams, and their speech equivalents, and examining the functional relevance of these vocal stimuli in communicating absolute strength and height to novel samples of listeners. To do this, we measured the upper-body strength and height of men and women and audio recorded them producing aggressive roars and distress screams as well as aggressive and distressed speech sentences. We then examined differences in the acoustic structure of these four types of voice recordings, and the effects of vocalizer height and strength on a range of acoustic parameters. Finally, to contrast the functional relevance of roars, screams, and their speech equivalents in communicating strength and size, we asked separate samples of listeners to estimate the strength or height of vocalizers from each type of vocal stimulus. Our key hypothesis was that the acoustic structure of vocal stimuli will reflect their function in accordance with motivational-structural rules, and thus, that the encoding and communication of strength and size will be maximized in aggressive and nonverbal speech variants.

## Experiment 1: Do roars and screams encode functional cues to strength and height?

In Experiment 1, we acoustically analyzed aggressive roars, distress screams, aggressive speech, and distressed speech, testing whether the acoustic structure of these vocal stimuli follows Morton’s motivational-structure rules, and whether it reliably predicts a vocalizer’s strength and height.

### Materials and methods

#### Participants

We audio recorded 61 adults (*M* age = 22.79 ± 1.12), who were 30 male and 31 female drama or acting students from the Royal Central School of Speech and Drama (London, United Kingdom) and the University of Sussex (Falmer, UK). Voice recordings and body measurements were collected from these participants as part of a broader research programme examining human vocal communication of strength (see also [12]). All participants provided informed consent and received monetary compensation in exchange for their participation. None were currently suffering from conditions that might affect their voice (e.g. colds, sore throats).

#### Procedure

All experiments were reviewed and approved by the University of Sussex’s Life Sciences & Psychology Cluster-based Research Ethics Committee (Sci-Tec C-REC) (Certificates of approval: ER/JR307/2, ER/JR307/4, ER/JR307/8), and comply with the American Psychological Association’s Ethical Principles of Psychologists and Code of Conduct.

***Voice recording*.** Vocalizations and speech sentences (*n* = 244) were recorded in a quiet room, with vocalizers standing 150 cm from a Zoom H4n microphone as demarked by a chair placed at this distance to restrict forward movement. In the aggressive context, vocalizers were instructed to imagine themselves in a battle or war scenario, about to charge and attack, and were instructed first to produce a given speech sentence imagining themselves in this context, and then a nonverbal vocalization expressing the same motivation [12]. In the distress context, vocalizers were asked to imagine that ‘the tables have turned’, and that they were now in a position of weakness, with an attacker charging at them, and again to produce a given speech sentence before producing an analogous nonverbal vocalization. Speech sentences were dictated by the experimenter and also displayed on a computer screen (Aggression context: ‘That’s enough, I’m coming for you!’; Distress context: ‘Please, show mercy, don’t hurt me!’).

Participants were encouraged to immerse themselves in each imagined context, to ‘let go of their inhibitions’, and to take as much time as they needed in order to obtain realistic vocal stimuli. They were also given the option not to vocalize if they felt that they could not naturally produce a given vocalization, and were permitted to repeat any sentence or vocalization until they were satisfied with their portrayal (see also [12]).

***Strength measurement*.** After vocalizing, participants’ heights were measured using metric tape. The average height of our sample was 182.03 ± 0.97 cm for men, and 167.10 ± 1.19 cm for women. Participants’ strength was assessed by measuring flexed bicep circumference, handgrip strength, and chest strength following previous studies [11,14,51]. These measures respectively explain 55%, 24% and 35% of the variance in strength among male college students as measured by weight-lifting machines [51].

To measure flexed bicep circumference (male *M* = 32.09 ± 0.60 cm; female *M* = 28.96 ± 0.70 cm), participants were instructed to rest the elbow of their dominant arm on a table while seated, to clench their fist, and to curl their forearm perpendicular to the table. The experimenter measured the circumference of the bicep at its highest point. A hydraulic hand dynamometer (Baseline standard) was used to measure handgrip strength (male *M* = 41.57 ± 1.36 kg; female *M* = 26.98 ± 1.06 kg) and chest strength (male *M* = 32.70 ±1.55 kg; female *M* = 19.12 ± 0.90 kg). We measured the handgrip strength of participants’ dominant arm with the instrument in its standard use (i.e. handle not inverted). To measure chest strength, the removable handle of the dynamometer was inverted, subjects grasped the handles, held the device to their chest with elbows extended and perpendicular to the body, and pressed the bars together as hard as possible with both hands [51].

Each strength measure was recorded twice per subject and the highest achievable score, representing greatest strength, was used in analyses. Strength measures were z-scored and then averaged to create a single strength score for each subject that weighted each strength measure equally (following [11,51]).

***Acoustic analysis*.** Vocal stimuli were analyzed using Praat 5.3.62 DSP package [52]. Recordings were saved as WAV files at 44.1 kHz sampling frequency and 16 bit amplitude resolution.

We used a dedicated batch-processing script containing four distinct procedures to measure a variety of acoustic parameters that have been implemented as potential vocal indicators of formidability in humans or other mammals, including parameters related to voice pitch (measured as fundamental frequency, *F*0), amplitude and intensity, noise and perturbation, and formants. The first procedure characterized fundamental frequency (F0), including mean F0, minimum F0, maximum F0, start-end F0 (a measure of the F0 contour), and F0CV (coefficient of variation over the duration of the signal, representing pitch variability). During visual inspection of each spectrogram, we also measured the proportion of the signal for which amplitude modulation was present, and created a measure representing this proportion as a percentage (%AM). We then applied two distinct smoothing algorithms to suppress either minor or major F0 fluctuations, and counted inflection points after each smoothing procedure, divided by the total duration of voiced segments, to derive two distinct indices of F0 modulation (inflex25—minor inflections, and inflex2—major inflections).

A second procedure measured mean amplitude and intensity contour (time of max intensity expressed as a percentage of the signal’s duration, and amplitude variability, intCV, representing the coefficient of variation of the intensity contour). A third procedure characterized noise and perturbation parameters, including harmonics-to-noise ratio (HNR, a measure of the ratio of harmonic spectral energy to chaotic spectral energy), jitter (small fluctuations in periodicity measured as the average of ‘local’, ‘rap’ and ‘ppq5’ measures in Praat) and shimmer (small changes in amplitude between consecutive periods, measured as the average of ‘local’, ‘apq5’ and ‘apq11’ parameters in Praat). While some researchers have argued that jitter and shimmer are inconsequential in the perception of non-pathological modal speech [53], these perturbation parameters appear to play a significant role in characterizing emotional nonverbal vocalizations. Indeed, acoustic analysis procedures similar to these have been applied successfully in previous studies of human babies’ cries [54,55].

A fourth and final procedure characterized the spectral centre of gravity for each vocal stimulus (spectral COG), calculated as the amplitude-weighted mean of signal frequencies. Given the acoustic structure of nonverbal vocalizations, particularly their high pitch, formant frequencies were poorly defined and difficult to measure via cepstrum or linear predictive coding analyses. However, the spectral centre of gravity carries some information about vocal tract resonances [56]. In addition, we measured the dominant frequency within sex-specific expected frequency ranges for the fourth formant, F4: 3108–4250 Hz for males, and 3524–4887 Hz for females [57]. These data have been used to establish formant thresholds in a previous study of vocal cues to upper-body strength [14]. This dominant formant frequency measure (hereafter ‘DFF4’) may be used as a proxy for vocal tract length, as articulatory manipulations of vocal tract shape minimally affect F4 [57], and as the measurement of dominant frequency within an expected F4 range is less likely to capture strong harmonics than for expected ranges of lower formants, as their amplitude declines exponentially with increasing frequency [48]. Importantly, F4 is among the strongest formant-based predictors of height in both men and women, explaining a similar amount of variance in height within-sexes as composite formant measures (e.g., formant spacing) and significantly more variance than F1, F2 or F3 [58].

**Fig 1:**
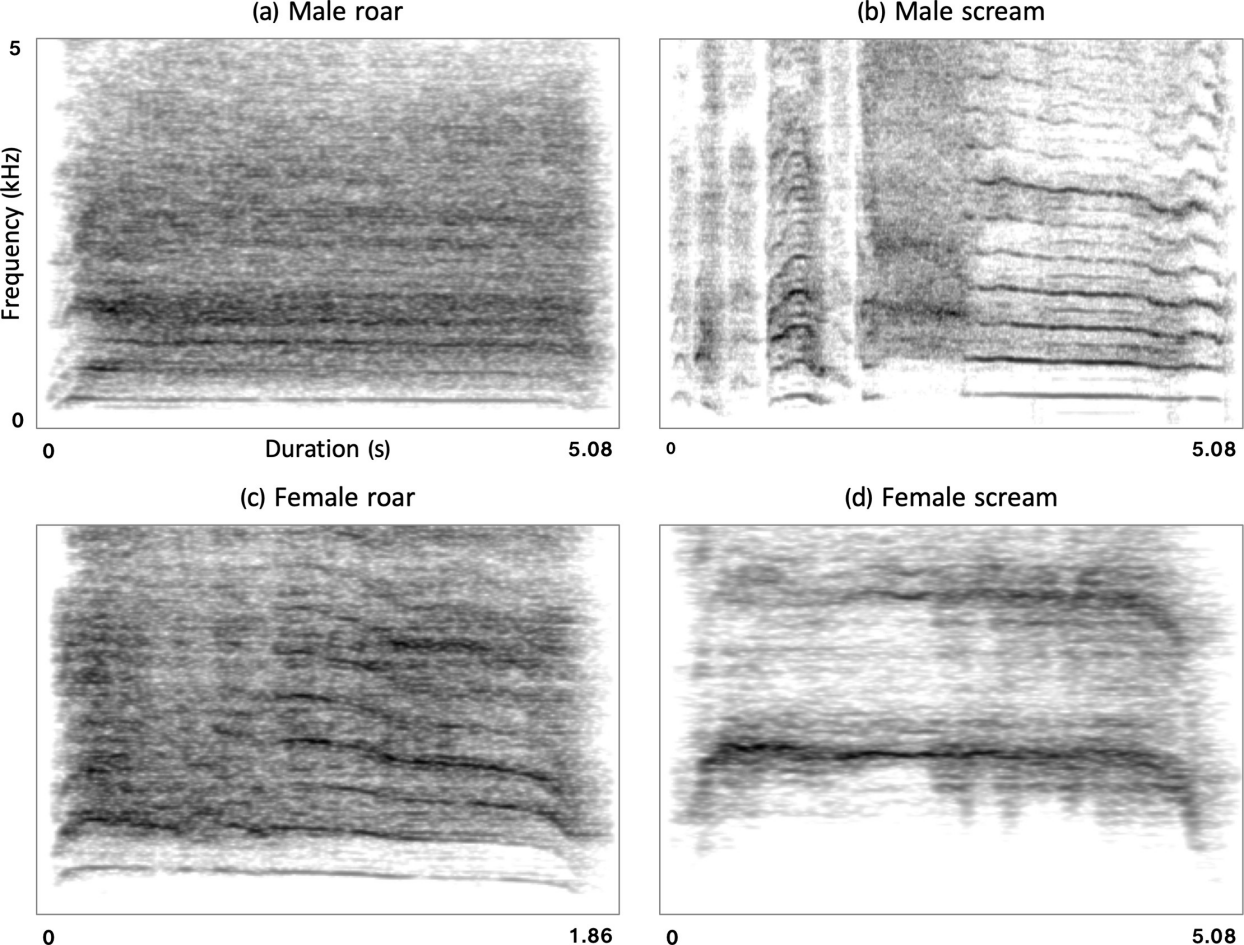
**Spectrograms illustrating the acoustic structure of a typical (a) male roar, (b) male scream, (c) female roar, and (d) female scream.** Note the higher F0 and more chaotic spectral structure of roars than screams.

Fig 1 presents spectrograms illustrating examplary roars and screams. For additional details regarding acoustic analysis, please refer to S1 Text.

#### Statistical analysis

To examine acoustic differences among vocal stimuli, we conducted a conventional leave-one-out discriminant function analysis (DFA) with forced entry, as this is less vulnerable to collinear variables, random effects, and type I errors than is stepwise entry [59]. We entered all acoustic variables except duration, using within-sex z-scores in place of raw measures for sexually dimorphic acoustic characteristics (mean F0, max F0, min F0, start-end F0, spectral COG, DFF4). We conducted a further DFA, split by sex, to investigate whether there were differences in the discriminability of vocal stimuli between sexes.

To investigate whether strength and height were encoded in the acoustic structure of vocal stimuli, we computed stepwise linear regressions with acoustic variables as predictors, and either actual strength or actual height as outcome variables, split by sex, stimulus type (speech/vocalization), and stimulus context (aggression/distress). Stepwise regressions were designed to test whether linear combinations of a wide set of acoustic characteristics could reliably predict physical formidability, and whether the structure of these models was consistent across stimulus types. To assess the individual contribution of each acoustic characteristic we computed zero-order correlations between each voice parameter and strength or height (reported in Supporting Information, S2 Text). The dataset for these analyses is also provided as Supporting Information (see S1 File).

### Results

#### Do roars, screams, and valenced speech sentences differ in acoustic structure?

**Fig 2:**
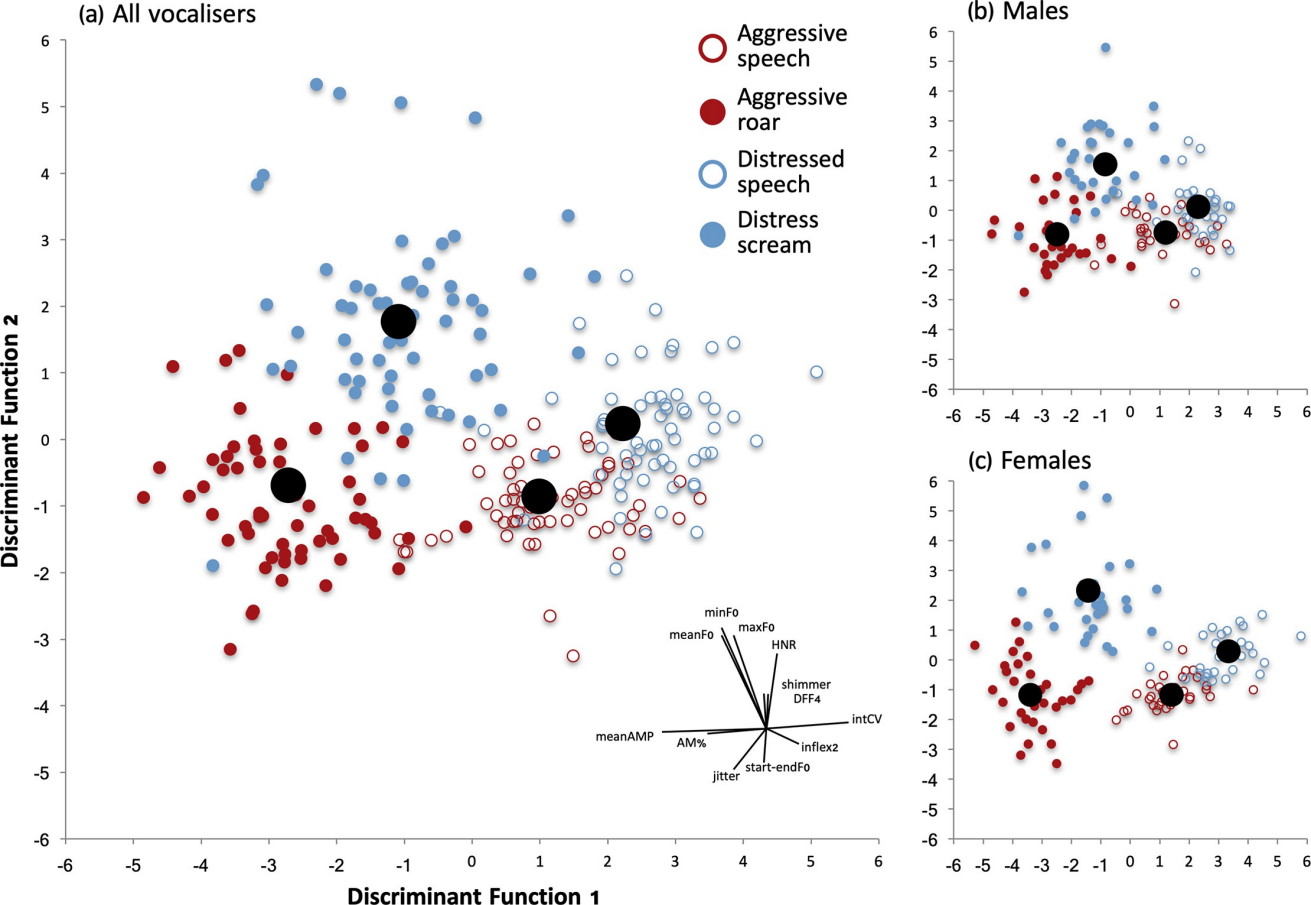
**Discriminant function analysis illustrating acoustic separation of voice conditions, (a) for all vocalizers, (b) for male vocalizers only, and (c) for female vocalizers only.** Each data point represents the centroid of a vocal stimulus as a function of the first two discriminant variables that maximize individual separation. Larger black circles represent mean group centroids for each voice condition. The radar plot on the bottom right of panel (a) represents the loadings of the acoustic variables on the first two discriminant functions. Mean amplitude, amplitude variability, and amplitude modulation were the main factors separating voice conditions on the first function (DF1, Table A in S1 Tables). The second function (DF2, Table A in S1 Tables) relied mostly on F0 and harmonics-to-noise ratio. The pattern of separation was similar in male (b) and female (c) vocalizers.

Discriminant function analyses indicated that all four voice conditions (roars, screams, aggressive speech, distress speech) were acoustically distinct (Fig 2). The DFA’s classification success rate significantly exceeded chance (correct classification = 79.9%, chance = 25%, *p* <0.0005). Supplementary tables report the factor loadings of acoustic parameters on the first three discriminant functions, collapsing across sexes (Table A in S1 Tables) and for male (Table B in S1 Tables) and female vocalizers (Table C in S1 Tables) separately (see S1 Tables, for all supplementary tables).

The first discriminant function (eigenvalue = 6.43, variance explained = 74.1%) differentiated each of the four voice conditions relatively equally while also separating nonverbal vocalizations from speech sentences (see Fig 2). Distressed speech stimuli were characterized as the quietest of the four voice conditions and had the greatest amplitude variability, the least amplitude modulation, and the most major F0 inflections, followed by aggressive speech and then distress screams. In contrast, roars were characterized by the highest amplitude, the least amplitude variability, the most amplitude modulation, and the fewest major F0 inflections.

The second discriminant function was less important in discriminating stimulus groups (eigenvalue = 1.93, variance explained = 22.2%), showing primarily that screams and, to a lesser degree, distressed speech sentences were more harmonic (high HNR) than were roars and aggressive speech (Figs 1 and 2). F0 variables (mean, max, min) loaded primarily on this function, but also on the first function. Mean values of measured acoustic variables (reported in Tables 1 and 2) showed that distress screams were characterized by the highest F0, followed by aggressive roars, with both speech conditions characterized by the lowest F0.

**Table 1:** Mean acoustic characteristics of male vocal stimuli. Figures in square brackets represent standard errors.

Acoustic Variable	Aggr. Speech		Aggr. Roar		Dist. Speech		Dist. Scream	
Duration (s)	1.92	*[0*.*07]*	1.27	*[0*.*12]*	2.66	*[0*.*14]*	1.35	*[0*.*17]*
Mean F0 (Hz)	311.6	*[10*.*96]*	378.7	*[7*.*53]*	288.5	*[11*.*96]*	466.9	*[25*.*50]*
Max F0 (Hz)	383.0	*[9*.*04]*	428.7	*[7*.*55]*	381.4	*[21*.*80]*	586.3	*[33*.*39]*
Min F0 (Hz)	213.3	*[9*.*17]*	273.2	*[11*.*12]*	204.8	*[9*.*89]*	333.8	*[15*.*06]*
Start–end F0 (Hz)	-1.62	*[12*.*85]*	31.76	*[12*.*21]*	-4.01	*[16*.*92]*	-21.64	*[23*.*99]*
F0 CV (Hz)	0.15	*[0*.*01]*	0.10	*[0*.*01]*	0.14	*[0*.*01]*	0.13	*[0*.*01]*
Minor F0 inflections	6.45	*[0*.*36]*	6.58	*[0*.*65]*	6.99	*[0*.*41]*	5.83	*[0*.*58]*
Major F0 inflections	0.88	*[0*.*06]*	0.62	*[0*.*09]*	0.94	*[0*.*08]*	0.60	*[0*.*07]*
Mean amplitude (dB)	62.57	*[0*.*94]*	71.94	*[0*.*70]*	56.39	*[1*.*02]*	67.40	*[0*.*84]*
Time of max intensity (%)	48.52	*[4*.*66]*	41.15	*[3*.*99]*	58.83	*[4*.*21]*	44.86	*[3*.*95]*
Intensity CV (dB)	1.43	*[0*.*05]*	0.81	*[0*.*05]*	1.53	*[0*.*05]*	1.05	*[0*.*06]*
Shimmer (dB)	0.14	*[0*.*003]*	0.68	*[0*.*35]*	0.66	*[0*.*36]*	1.47	*[0*.*51]*
Jitter (Hz)	0.018	*[0*.*001]*	0.029	*[0*.*002]*	0.017	*[0*.*001]*	0.019	*[0*.*002]*
HNR (dB)	7.36	*[0*.*42]*	5.51	*[0*.*73]*	9.26	*[0*.*48]*	10.13	*[0*.*81]*
Amplitude modulation (%)	24.02	*[3*.*05]*	60.99	*[3*.*76]*	11.50	*[2*.*64]*	33.81	*[4*.*35]*
Centre of gravity (Hz)	1000.3	*[37*.*28]*	1143.4	*[30*.*68]*	842.2	*[41*.*21]*	1085.2	*[51*.*54]*
Dominant formant frequency DFF4 (Hz)	3381.8	*[43*.*53]*	3314.5	*[40*.*14]*	3438.3	*[45*.*71]*	3508.3	*[57*.*68]*

**Table 2:** Mean acoustic characteristics of female vocal stimuli. Figures in square brackets represent standard errors.

Acoustic Variable	Aggr. Speech		Aggr. Roar		Dist. Speech		Dist. Scream	
Duration (s)	1.98	*[0*.*08]*	1.21	*[0*.*12]*	2.54	*[0*.*12]*	1.16	*[0*.*09]*
Mean F0 (Hz)	437.1	*[14*.*05]*	620.2	*[33*.*93]*	420.8	*[14*.*06]*	898.6	*[65*.*27]*
Max F0 (Hz)	568.7	*[16*.*57]*	767.4	*[59*.*56]*	557.4	*[21*.*50]*	1087.7	*[70*.*06]*
Min F0 (Hz)	259.3	*[12*.*11]*	398.4	*[21*.*96]*	314.0	*[12*.*22]*	614.4	*[43*.*07]*
Start–end F0 (Hz)	107.1	*[20*.*47]*	62.14	*[54*.*04]*	5.56	*[19*.*58]*	-42.36	*[36*.*19]*
F0 CV (Hz)	0.17	*[0*.*01]*	0.14	*[0*.*02]*	0.13	*[0*.*01]*	0.14	*[0*.*01]*
Minor F0 inflections	6.37	*[0*.*33]*	5.41	*[0*.*80]*	8.09	*[0*.*42]*	6.41	*[0*.*49]*
Major F0 inflections	0.81	*[0*.*07]*	0.56	*[0*.*08]*	1.02	*[0*.*08]*	0.57	*[0*.*06]*
Mean amplitude (dB)	61.11	*[0*.*91]*	73.97	*[0*.*69]*	53.35	*[1*.*21]*	68.24	*[0*.*99]*
Time of max intensity (%)	38.58	*[4*.*19]*	39.39	*[3*.*88]*	59.36	*[4*.*41]*	43.60	*[4*.*16]*
Intensity CV (dB)	1.42	*[0*.*04]*	0.76	*[0*.*03]*	1.43	*[0*.*05]*	0.94	*[0*.*05]*
Shimmer (dB)	0.44	*[0*.*30]*	1.58	*[0*.*56]*	2.10	*[0*.*67]*	2.86	*[0*.*67]*
Jitter (Hz)	0.018	*[0*.*001]*	0.026	*[0*.*003]*	0.014	*[0*.*001]*	0.015	*[0*.*002]*
HNR (dB)	8.36	*[0*.*43]*	7.85	*[1*.*14]*	10.56	*[0*.*44]*	14.02	*[0*.*97]*
Amplitude modulation (%)	28.42	*[3*.*19]*	48.04	*[5*.*17]*	14.52	*[1*.*89]*	46.48	*[3*.*79]*
Centre of gravity (Hz)	1321.2	*[44*.*60]*	1411.8	*[43*.*43]*	1156.5	*[63*.*72]*	1413.5	*[55*.*91]*
Dominant formant frequency DFF4 (Hz)	3763.3	*[52*.*94]*	3789.6	*[57*.*27]*	3881.5	*[59*.*02]*	3947.1	*[81*.*69]*

Finally, aggressive roars displayed higher jitter than did all other stimuli, whereas screams (but not distressed speech) were characterized by higher shimmer and a higher dominant formant frequency (DFF4) than aggressive stimuli. We excluded duration from our discriminant analyses because multiple-word speech sentences were inherently longer than single vocalizations, but we report duration means for each voice condition (see Tables 1 and 2). The acoustic characteristics separating vocal stimuli were similar across sexes (Fig 2, see also Tables B and C in S1 Tables).

#### Do roars, screams and valenced speech stimuli contain acoustic cues to actual strength and height?

Strength did not correlate with height among either male (*r* = -.04, *p* = .833) or female (*r* = .083, *p* = .655) vocalizers. Therefore, at least in our sample, these two physical measurements appear to represent distinct aspects of physical formidability.

We observed very few significant, systematic relationships between acoustic variables and vocalizer height or strength (see Tables D and E in S1 Tables). The only notable exception was that the dominant formant frequency (DFF4) was negatively associated with strength for female vocalizers in all voice stimulus types except distress screams (Table D in S1 Tables). Zero-order correlations corroborated the absence of systematic acoustic predictors of strength and height (see S2 Text).

### Discussion

The high classification accuracy of the discriminant function analysis shows that vocal stimuli were characterized by distinct acoustic structures that varied according to both stimulus type (speech/nonverbal vocalization) and context (aggression/distress). Nonverbal emotional expressions of anger and fear have, in earlier DFA’s, been confused [60], offering a partial explanation for the slight overlap among speech categories in the present DFA.

Nonverbal vocalizations displayed more variability in acoustic characteristics, were louder, higher-pitched, and exhibited more amplitude modulation than did their speech equivalents, consistent with evidence that laughter exhibits higher F0 mean and range [61] and higher F1 [62] compared to speech. This could be due to a lack of linguistic constraints on nonverbal vocalizations [63] enabling a wider acoustic space compared to speech. Indeed, speech necessitates a relatively low pitch/spectral density for formant perception [64] and places constraints on intonation for semantic encoding [65] and phoneme recognition [66].

The co-occurrence of high F0, high amplitude, and nonlinear phenomena in nonverbal vocalizations suggests that they were produced with high vocal effort [67]. Fundamental frequency and amplitude are both known to increase with subglottal pressure [68,69], and nonlinear phenomena (indicating a transition to unstable regimes of vocal fold vibration) arise more commonly when subglottal pressure is relatively high [69–73]. By operating at or near the upper limits of amplitude production, nonverbal vocalizations may be more readily subject to anatomical constraints that constrain vocal exaggeration and thus increase the honesty of acoustic indexical cues [44,45,47], and thus, may communicate physical traits of the vocalizer more effectively than speech. This may be particularly true of aggressive roars, which exhibited the most nonlinearities of all stimuli.

In accordance with motivational-structural rules [30,31,33], distress stimuli were more tonal (higher HNR and less amplitude modulation) than aggressive stimuli. In nonhuman mammals, distress vocalizations are indeed typically tonal, but may be noisy if fear and aggression are conflicting or if their function is to solicit support from distant allies [33,74]. Our analyses showed that roars and screams occupied opposite extremes in terms of harmonics-to-noise ratio, again suggesting that vocalizations exploit wider ranges of acoustic space compared to speech utterances, which fell in between these extremes. Screams were characterized by a higher F0 (see Fig 1), lower jitter, and a higher dominant formant frequency (DFF4) than roars, also as predicted by motivational-structural rules. Yet these differences were not observed between aggressive and distressed speech. Our results therefore suggest that the acoustic constraints necessary to intelligibly communicate speech may limit the expression of motivational-structural rules in speech, including emotional or valenced speech.

Reliable cues to height were not consistently encoded in the acoustic structure of our vocal stimuli. While previous work has shown that formant frequencies in modal speech predict vocal tract length and thus height within sexes [58], the prevalence of high pitch/low spectral density and/or amplitude modulation in nonverbal vocalizations resulted in poor representation of vocal tract resonances. This was also observed to some extent in valenced speech sentences that were also produced with high vocal effort, potentially explaining why our formant-based voice parameters (COG, DFF4) did not reliably predict height even in speech. This result may also reflect variation in vocalizers’ propensity to exaggerate size in an aggressive context or minimize size in a distress context.

Although formants are a well-established indicator of human height [58], previous research has produced inconsistent findings regarding the acoustic encoding of physical strength in speech [11,13,14]. Formant dispersion has been reported to predict male strength [13,14], but only in cases where correlations between height and strength were strong [13,14], suggesting that any relationship between strength and formants is mediated by the relationship between height and formants. However, the unexpected but consistent association between DFF4 and strength in our sample of females suggests that spectral characteristics reflecting complex contributions of both source and filter may still play a role in encoding strength.

While the present study utilized an amalgamated strength measure based on flexed bicep circumference, handgrip strength, and chest strength (following [11]), some other studies examining vocal correlates of strength have utilized amalgamated scores based on fewer measures (e.g., flexed bicep circumference and handgrip strength only [12,15]), or have examined strength measures individually (e.g., biceps only, handgrip strength only [14,16]). Nevertheless, different measures of upper-body strength covary within and between individuals and, given that these previous studies likewise did not report consistent or robust acoustic correlates of strength, differences in how strength was computed across these few studies are not likely to explain such null results.

To summarize, despite indications that our aggressive roars and distress screams utilised a wider acoustic space than did speech sentences, and despite measuring a much wider set of acoustic variables than previous studies examining cues to strength in speech [11,13,14], our investigations still failed to reveal consistent acoustic cues to strength. Thus, despite one study that reported an association between F0 and strength [13] in speech, our study corroborates the more commonly observed lack of significant relationship between F0 and strength in the human voice [11,14]. Thus, while our results support the general hypothesis that aggressive roars and distress screams are acoustically distinct and evolved to respectively maximize or minimize the impression of strength and threat, their acoustic structure did not reliably predict vocalizer strength or height within call types.

## Experiments 2 and 3: Can listeners estimate strength and height from roars, screams and valenced speech?

Following acoustic analysis, we used playback experiments to assess the functional relevance of aggressive roars, aggressive speech, distress screams, and distressed speech in communicating strength and body size. Separate samples of listeners judged either the physical strength or height of the vocalizers whose voices we analyzed in Experiment 1.

We predicted that ratings of strength and height would be highest for aggressive stimuli, as such vocalizations index quantitative information regarding the severity of potential threat (i.e. the formidability of the aggressor), potentially adaptively influencing decision-making in competitive interactions. In contrast, for distress stimuli, listeners may have been selected to pay attention to the level of distress rather than to the signaller’s formidability. Indeed, among nonhuman mammals, vocalizations produced in aggressive contexts function specifically to signal formidability, and in these contexts many species functionally exaggerate acoustic cues to dominance and size [47,75–78].

Male-male competition is thought to have played a key role in shaping men’s vocal signals [79,80] and in producing sexually dimorphic acoustic features that function in part to more effectively communicate threat potential in men’s than women’s voices. Hence, we further predicted that listeners would more accurately estimate strength and height from male than female speech stimuli. However, as size and strength are relevant in both mate competition and mate choice contexts, we did not predict sex differences in listeners’ judgments of strength.

## Materials and methods

### Participants

Participants from the USA were recruited from Amazon Mechanical Turk (see [81] for a review of the validity of this research method) to provide voice-based assessments of strength and height. All participants provided informed consent and completed the experiments online using a custom computer interface. They were compensated with $3.50 USD. Ninety adults took part in Experiment 2 (48 females and 42 males, age = 33.82 ± 9.60) and 60 different adults took part in Experiment 3 (30 females and 30 males, age = 33.80 ± 8.98). Data from four participants in Experiment 2 and six participants in Experiment 3 who did not complete the experiment but rated more than half of the stimuli were included in analyses, as the exclusion of their responses did not change the overall pattern of results.

#### Voice stimuli

Participants rated all 244 voice stimuli acquired in Experiment 1 (61 vocalizers x 4 stimulus types) on one dimension (either strength or height). To reliably assess the effect of amplitude on listeners’ attributions, it was necessary for listeners to maintain the same listening volume for the duration of the playback experiment. The difference in mean amplitude between the quietest (40.40 dB) and loudest (81.66 dB) stimulus was large; hence, we partially normalized amplitude to minimize auditory discomfort while ensuring that listeners could clearly hear all stimuli. Speech stimuli (mean amplitude = 58.31 dB) were consistently quieter than vocalizations across sexes (70.27 dB), therefore, we increased the amplitude of speech stimuli and decreased the amplitude of vocalizations by 4 dB each.

#### Procedure

Playback studies were hosted in *Syntoolkit*, a dedicated online testing platform used to generate and present psychology studies (see e.g., [82]). Participants were directed to the URL testing site and provided informed consent before beginning the study. They were instructed to use headphones and to complete the experiment in a quiet place. Listeners heard a demo sound file before commencing the experiment which contained the loudest stimulus and the fifth quietest stimulus, and were instructed to raise their volume until they could clearly hear the quiet vocalization while the loudest vocalization did not cause discomfort. Following this, listeners were asked not to adjust the volume during the experiment unless it became too uncomfortable. Listeners were also asked at the end of the experiment if they had adjusted their volume at any point. Due to the agonistic nature of the stimuli, they were made aware that if they felt uncomfortable or distressed listening to the sounds, they could stop the experiment.

Voice stimuli were blocked by sex (male/female), stimulus type (speech/vocalization), and stimulus context (aggression/distress). The order of blocks and stimuli within blocks was randomized. Before each block, participants were reminded to listen to each stimulus in full before rating it, and informed that they could take a break at any time. Listeners rated the physical strength (Experiment 2) or height (Experiment 3) of each voice stimulus (“Rate how strong/tall this vocalizer is”) on a 101-point scale from 0 (extremely weak/short) to 100 (extremely strong/tall).

Listeners were debriefed upon completion that the roars and screams were acted, and that the vocalizers were not really experiencing aggression or distress. We inspected listeners’ ratings and compared their reaction times against stimulus duration to ensure that they completed the experiments properly. Data from two participants who did not do so were removed (and are not reported in the participant statistics given above).

#### Statistical analysis

In a series of linear mixed models, we first tested whether male vocalizers were stronger/taller than female vocalizers. Next, we tested the effects of vocalizer sex, listener sex, stimulus context, and stimulus type on attributed strength/height ratings. The third set of models added actual strength/height into the previous models to assess accuracy in listeners’ strength and height estimates. As the strength and height distributions for males and females displayed little overlap, we split these models by vocalizer sex rather than including sex as a factor. In all models, we included listener identity as a subject variable and vocalizer identity as a random factor, thus allowing the intercepts and slopes of the relationships between predictors and outcomes to vary between both vocalizers and listeners and testing null hypotheses based on the average of these intercepts and slopes.

Effect sizes were estimated using *R*^*2*^ coefficients derived from simple linear regressions among relevant variables, and using ***γ*** coefficients derived from the linear mixed models. *R*^*2*^ values denote the percentage of variance in mean strength ratings explained by variance in actual strength, and can be interpreted as representing the overall reliability of listeners’ strength estimations, adjusted to the linear sensitivity of listeners to variation in actual strength within each condition. Differences in slope gradients between conditions, represented by the gamma (***γ***) statistic denoting the standardised increase in rated strength/height per one unit increase in actual strength/height, indicate linear differences in listeners’ sensitivity to variation in vocalizer strength or height.

Subsequently, we computed stepwise linear multiple regressions to assess relationships between acoustic characteristics and strength/height ratings. The previously measured acoustic variables were used as predictors, and either mean strength or mean height ratings as outcome variables. Participants who indicating having modified their volume during the experiment (Experiment 1: *n* = 4, Experiment 2: *n* = 15) were excluded from the calculation of mean ratings, enabling valid analysis of the effect of amplitude on ratings. Regression models were split by sex, stimulus type (speech/vocalization), and stimulus context (aggression/distress).

### Results

#### Do stimulus context and type affect ratings of strength and height?

**Fig 3:**
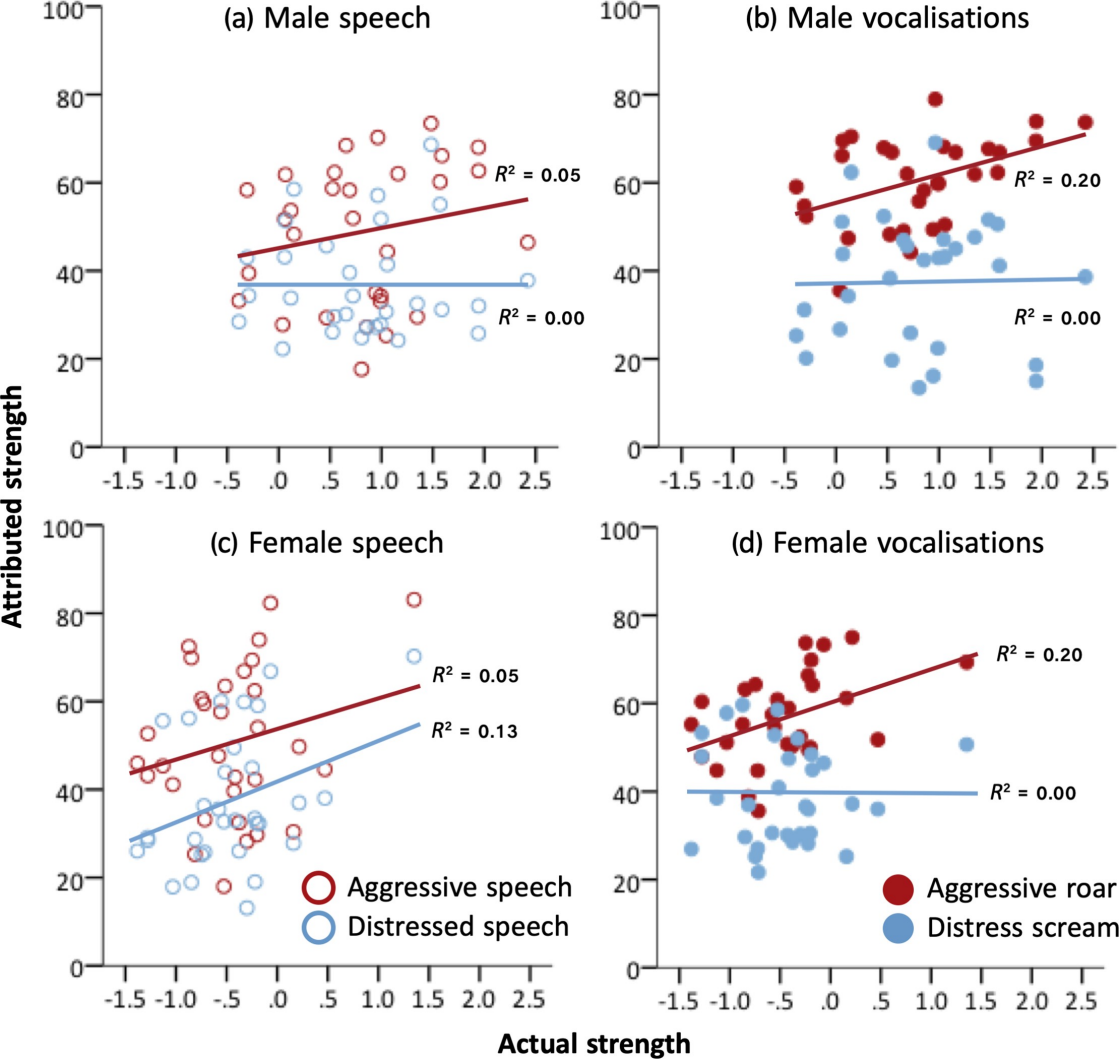
**Attributed strength as a function of actual strength, when listeners rated (a) male speech stimuli, (b) male vocalizations, (c) female speech stimuli, and (d) female vocalizations.** Each data point represents the mean strength rating averaged across listeners attributed to each vocalization. Blue circles represent distress stimuli, red circles represent aggressive stimuli. Open circles represent speech stimuli, closed circles represent vocalizations. *R*^*2*^ values for each regression line are reported in the graphs. Removing the strongest female vocalizer from our analyzes did not affect the significance of our results.

***Strength attributions*.** On average, aggressive stimuli were rated as stronger (*M* = 54.15 ± 0.75) than distress stimuli (*M* = 37.84 ± 0.75, Fig 3, Table 3, *p* < .0005). This difference was significantly larger when listeners rated nonverbal vocalizations (roars vs. screams: *M* difference = 20.31) than when they rated speech sentences (*M* difference = 12.31, Fig 3, Table 3, *p* < .0005; except when male listeners rated female vocalizers, Table 3, *p* < .001).

**Table 3:** Strength attributions: Linear mixed model testing the effects of vocalizer sex, listener sex, stimulus context, and stimulus type on rated strength.

Source	*df*_*1*_, *df*_*2*_	*F*	*p*
i. Intercept	1, 88.01	3892.10	**< .001**
ii. Vocalizer sex	1, 5398.65	0.00	.970
iii. Listener sex	1, 88.01	0.06	.813
iv. Stimulus context	1, 16376.86	2940.38	**< .001**
v. Stimulus type	1, 16376.86	285.87	**< .001**
vi. Vocalizer sex x listener sex	1, 5398.65	0.02	.876
vii. Vocalizer sex x stimulus context	1, 16390.45	9.33	**.002**
viii. Vocalizer sex x stimulus type	1, 16390.45	13.96	**< .001**
ix. Listener sex x stimulus context	1, 16376.86	1.20	.273
x. Listener sex x stimulus type	1, 16376.86	0.21	.648
xi. Stimulus context x stimulus type	1, 16376.86	176.99	**< .001**
xii. Voc sex x list sex x stimulus context	1, 16390.45	3.38	.066
xiii. Voc sex x list sex x stimulus type	1, 16390.45	0.01	.921
xiv. Voc sex x stimulus context x stimulus type	1, 16390.45	33.17	**< .001**
xv. List sex x stimulus context x stimulus type	1, 16376.86	7.22	**.007**
xvi. Voc sex x list sex x stim context x stim type	1, 16390.45	4.58	**.032**

**Fig 4:**
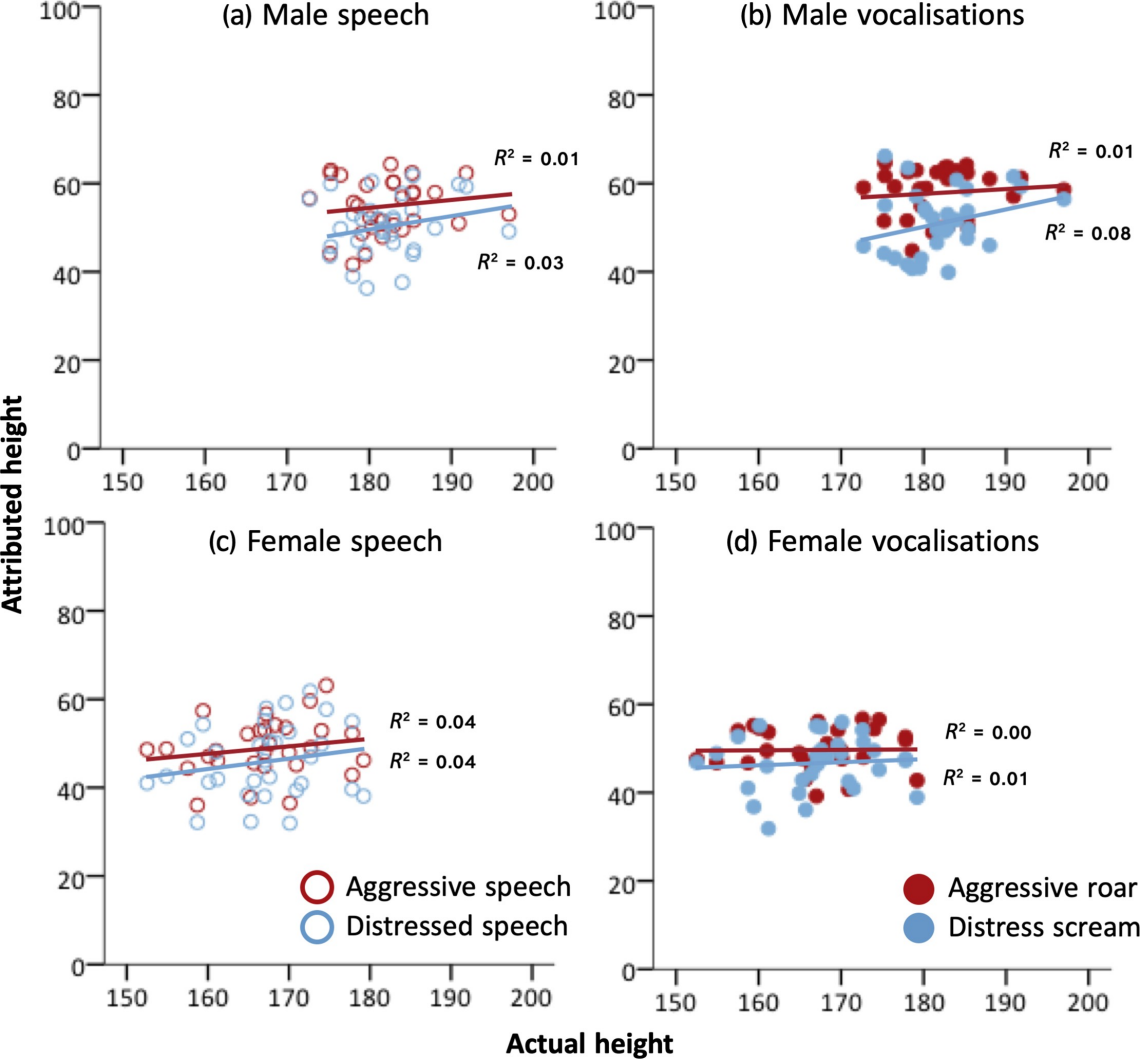
**Attributed height as a function of actual height, when listeners rated (a) male speech stimuli, (b) male vocalizations, (c) female speech stimuli, and (d) female vocalizations.** Each data point represents the mean height rating averaged across listeners attributed to each vocalization. Blue circles represent distress stimuli, red circles represent aggressive stimuli. Open circles represent speech stimuli, closed circles represent vocalizations. *R*^*2*^ values for each regression line are reported in the graphs.

***Height attributions*.** Vocalizers were rated as taller when producing aggressive than distressed sounds and sentences. This was particularly true for male vocalizers (*M* difference = 5.44 vs. *M* female vocalizers = 2.91, Fig 4, Table 4, *p* < .001; see Table 5 for strength attributions), and female raters (*M* difference = 5.98 vs. *M* difference in other voice conditions = 3.61, Table 6, *p* = .046). Speech sentences and nonverbal vocalizations generally elicited similar height ratings, except when female listeners rated aggressive stimuli, in which case they rated vocalizers as taller when producing roars (*M* = 56.16 ± 0.74) than when producing aggressive speech (*M* = 52.75 ± 0.73, *M* difference = 3.41, *M* difference other voice conditions = 0.48, Table 4, *p* = .046).

**Table 4:** Height attributions: Linear mixed model testing the effects of vocalizer sex, listener sex, stimulus context, and stimulus type on rated height.

Source	*df*_*1*_, *df*_*2*_	*F*	*p*
i. Intercept	1, 58.16	11922.30	**< .001**
ii. Vocalizer sex	1, 3618.53	279.44	**< .001**
iii. Listener sex	1, 58.16	8.34	**.005**
iv. Stimulus context	1, 10577.56	234.15	**< .001**
v. Stimulus type	1, 10476.98	19.87	**< .001**
vi. Vocalizer sex x listener sex	1, 3618.53	1.82	.177
vii. Vocalizer sex x stimulus context	1, 10578.54	21.54	**< .001**
viii. Vocalizer sex x stimulus type	1, 10421.61	6.91	**.009**
ix. Listener sex x stimulus context	1, 10577.56	5.60	**.018**
x. Listener sex x stimulus type	1, 10476.98	14.38	**< .001**
xi. Stimulus context x stimulus type	1, 10432.64	5.20	**.023**
xii. Voc sex x list sex x stimulus context	1, 10578.54	0.17	.684
xiii. Voc sex x list sex x stimulus type	1, 10421.61	0.92	.339
xiv. Voc sex x stimulus context x stimulus type	1, 10406.88	3.81	.051
xv. List sex x stimulus context x stimulus type	1, 10432.64	3.97	**.046**
xvi. Voc sex x list sex x stim context x stim type	1, 10406.88	3.81	.051

**Table 5:** Strength estimation: Linear mixed models testing the effects of actual strength, stimulus context, stimulus type, and listener sex on the rated strength of females and males.

Source	Females			Males		
	*df*_*1*_, *df*_*2*_	*F*	*p*	*df*_*1*_, *df*_*2*_	*F*	*p*
i. Intercept	1, 110.71	3159.40	**< .001**	1, 106.86	2814.58	**< .001**
ii. Actual strength	1, 2697.52	162.96	**< .001**	1, 606.93	55.03	**< .001**
iii. Stimulus context	1, 8309.89	706.95	**< .001**	1, 8063.01	598.41	**< .001**
iv. Stimulus type	1, 8309.89	2.70	.100	1, 8063.01	99.14	**< .001**
v. Listener sex	1, 110.71	0.21	.651	1, 106.86	0.06	.810
vi. Strength x stimulus context	1, 8317.01	9.80	**.002**	1, 8066.40	80.17	**< .001**
vii. Strength x stimulus type	1, 8317.01	38.67	**< .001**	1, 8066.40	2.35	.126
viii. Strength x listener sex	1, 2697.52	0.42	.515	1, 2606.93	0.05	.826
ix. Stim context x stim type	1, 8309.89	77.82	**< .001**	1, 8063.01	88.97	**< .001**
x. Stim context x listener sex	1, 8309.89	2.12	.145	1, 8063.01	0.45	.502
xi. Stim type x listener sex	1, 8309.89	1.47	.226	1, 8063.01	0.10	.749
xii. Strength x stimulus context x stimulus type	1, 8317.01	50.25	**< .001**	1, 8066.40	1.15	.284
xiii. Strength x stimulus context x listener sex	1, 8317.01	0.01	.910	1, 8066.40	0.16	.686
xiv. Strength x stimulus type x listener sex	1, 8317.01	1.72	.190	1, 8066.40	0.04	.851
xv. Stimulus context x stimulus type x listener sex	1, 8309.89	11.32	**.001**	1, 8063.01	1.80	.180
xvi. Strength x stimulus context x stimulus type x listener sex	1, 8317.01	2.20	.138	1, 8066.40	2.41	.120

**Table 6:** Height estimation: Linear mixed models testing the effects of actual height, stimulus context, stimulus type, and listener sex on the rated height of females and males.

Source	Females			Males		
	*df*_*1*_, *df*_*2*_	*F*	*p*	*df*_*1*_, *df*_*2*_	*F*	*p*
i. Intercept	1, 1782.63	18.64	**< .001**	1, 1727.83	6.30	**.012**
ii. Actual height	1, 1751.63	13.45	**< .001**	1, 1713.07	16.08	**< .001**
iii. Stimulus context	1, 5286.69	2.15	.143	1, 5154.25	9.29	**.002**
iv. Stimulus type	1, 5294.85	7.66	**.006**	1, 5155.61	0.95	.331
v. Listener sex	1, 1782.63	.32	.571	1, 1727.83	0.03	.855
vi. Height x stimulus context	1, 5291.60	1.36	.244	1, 5154.25	6.95	**.008**
vii. Height x stimulus type	1, 5294.95	7.38	**.007**	1, 5155.62	1.24	.265
viii. Height x listener sex	1, 1751.63	0.09	.761	1, 1713.07	0.00	.956
ix. Stim context x stim type	1, 5251.09	0.02	.888	1, 5155.61	2.73	.099
x. Stim context x listener sex	1, 5286.69	.73	.391	1, 5154.25	0.03	.858
xi. Stim type x listener sex	1, 5294.85	1.11	.293	1, 5155.61	0.37	.542
xii. Height x stimulus context x stimulus type	1, 5251.18	0.02	.897	1, 5155.62	2.44	.118
xiii. Height x stimulus context x listener sex	1, 5291.60	0.83	.362	1, 5154.25	0.02	.901
xiv. Height x stimulus type x listener sex	1, 5294.95	0.85	.357	1, 5155.62	0.30	.582
xv. Stimulus context x stimulus type x listener sex	1, 5251.09	.11	.743	1, 5155.61	0.29	.593
xvi. Height x stimulus context x stimulus type x listener sex	1, 5251.18	.11	.742	1, 5155.62	0.38	.540

#### Are there sex differences in actual or rated strength and height?

***Effect of vocaliser sex*.** Linear mixed model analysis revealed that males (*M* = 0.81 ± 0.11) were physically stronger than females (*M* = -0.46 ± 0.11, *F*_(1, 61)_ = 64.83, *p* < .0005), and taller (*M* = 182.03 ± 1.09 cm) than females (*M* = 166.94 ± 1.04 cm, *F*_(1, 61)_ = 101.02, *p* < .0005). Yet, males were only rated as stronger than females by male listeners judging aggressive roars (Table 3, *p* = .032). For all other conditions, females were rated as comparably strong as males (Fig 3), indicating that listeners’ strength attributions were generally not consistent with sexual dimorphism in actual strength.

Height ratings were consistent with sexual dimorphism in height. Listeners rated males as taller than females across all stimulus types and contexts (Fig 4, Table 4, *p* < .0005). This sex difference in height ratings was larger for aggressive (*M* difference = 7.04) than distress stimuli (*M* difference = 4.51, Table 6, *p* < .0005), and for nonverbal vocalizations (*M* difference = 6.50) than for speech sentences (*M* difference = 5.06, Table 4, *p* = .009).

***Effect of listener sex*.** Female listeners rated aggressive roars produced by female vocalisers as stronger than did male listeners (*M* difference = 2.58, Table 3, *p* = .032), but otherwise produced comparable strength ratings (*M* difference for other voice conditions = 0.37). Female listeners (*M* = 52.04 ± 0.66) generally judged vocalisers as taller than did male listeners (*M* = 49.36 ± 0.66, Table 4, *p* = .005), particularly when listening to aggressive roars (*M* difference = 4.9, *M* difference other voice conditions = 1.94, Table 6, *p* = .046).

#### Can listeners accurately estimate strength and height from the voice?

***Strength estimation*.** For male vocalizers, actual strength predicted attributed strength only when listeners rated aggressive stimuli (Table 5, *p* < .001). For female vocalizers, listeners could estimate strength from aggressive roars, aggressive speech, and distressed speech, but not distress screams (Table 5, *p* < .001; see also ***γ*** statistics in Table 7 denoting the standardised increase in rated strength per one unit increase in actual strength). For both male and female vocalizers, the reliability of strength estimation was higher for aggressive roars than for aggressive speech or female distressed speech (Fig 3; refer to *R*^*2*^ denoting variance in mean strength ratings explained by actual strength). Thus, listeners consistently estimated strength from aggressive but not distress stimuli, and estimated strength most reliably from aggressive roars.

**Table 7:** Standardised linear mixed model coefficients representing the sensitivity of listeners to variation in vocalizer strength and height. Each coefficient represents the average of listeners’ individual slopes for the relationship between actual strength/height and attributed strength/height. Significances represent whether each average slope was significantly different from zero. Separate models are reported for male and female vocalizers.

Source	Females		Males	
	*γ*	*p*	*γ*	*p*
**Strength**				
Aggressive speech	.18	**< .001**	.15	**< .001**
Distressed speech	.24	**< .001**	.01	.283
Aggressive roar	.20	**< .001**	.20	**< .001**
Distress scream	-.03	.198	.02	.379
**Height**				
Aggressive speech	.07	**.003**	.03	.171
Distressed speech	.09	**< .001**	.05	**.021**
Aggressive roar	.01	.749	.02	.270
Distress scream	.03	.140	.11	**< .001**

There was little evidence for listener sex or vocalizer sex differences in the capacity to estimate strength. The only exception was for distressed speech, whereby listeners were more sensitive to variation in actual strength when rating female than male vocalizers.

***Height estimation*.** For male vocalizers, actual height predicted rated height when listeners rated distress stimuli but not aggressive stimuli (Fig 4, Table 6, *p* = .008; see also Table 7 for ***γ*** effect sizes). For female vocalizers, actual height predicted attributed height when listeners rated speech stimuli but not nonverbal vocalizations (Fig 4, Table 6, *p* = .007; see Table 7 for ***γ***). Effect sizes for the relationship between actual and attributed height were much smaller than those for the relationship between actual and attributed strength (Figs 3 and 4).

As with strength, there were few sex differences in height estimation, except that listeners were more sensitive to variation in actual strength in male than female vocalizers when rating distress screams.

#### Are ratings of physical traits related to acoustic characteristics?

Mean amplitude consistently predicted ratings of physical strength across stimulus categories and sexes (see Tables F and G in S1 Tables). In addition, vocalizers who were rated as stronger generally produced rougher voice stimuli. Decreases in F0 variability, and increases in amplitude modulation and duration with rated strength were also observed, though inconsistently (Table F in S1 Tables). Zero-order correlations corroborated the influence of these acoustic characteristics on rated strength (see S2 Text).

The influence of acoustic characteristics on height ratings was in general much less consistent than for strength ratings (Table G in S1 Tables). In males, louder and lower-pitched stimuli were consistently judged as produced by taller vocalizers. Male roars and screams characterized by higher jitter were also rated as produced by taller vocalizers. No acoustic characteristic consistently predicted height ratings of female vocalizers, but louder aggressive roars and distressed speech were rated as produced by taller vocalizers. Zero-order correlations corroborated the lack of consistent acoustic predictors of rated height (S2 Text).

### Discussion

The results of playback experiments indicated that roars maximized impressions of strength relative to other vocal stimuli. Listeners attributed higher strength and height ratings to aggressive stimuli (aggressive speech and roars) than to distress stimuli (distress speech and screams), consistent with functional exaggeration of acoustic cues to body size by nonhuman mammals in aggressive contexts [47,75–78]. This effect may be due to acoustic differences between stimuli: aggressive roars were characterized by higher roughness and amplitude than distress screams, as well as a lower F0 and DFF4. This suggests that aggressive roars capitalised on perceptual associations between low frequency sounds and large size, exaggerating perceived formidability relative to distress screams, which instead exploited perceptual associations between high frequencies and small size or submission [9,11,31,33].

In the absence of differences in F0 and DFF4 between aggressive and distressed speech, the smaller difference in strength ratings between these speech stimuli (compared to roars and screams) may be attributed to differences in roughness and amplitude, consistent with the observation that both roughness and amplitude consistently predicted listeners’ ratings within voice conditions. Differences in the linguistic content of aggressive and distressed speech may have also contributed to differences in listeners’ ratings between the two types of speech stimuli. The verbal content of each speech stimulus was selected specifically to convey either aggression (That’s enough, I’m coming for you!) or distress (‘Please, show mercy, don’t hurt me!), as previous studies have failed to find acoustic correlates of actual or perceived strength in emotionally neutral speech [11,16]. Nevertheless, a third speech condition, in which participants produce the same linguistic content while imagining themselves in each of the aggressive and distress situations, may reduce the ecological validity of the task but could in turn help to disentangle the influence of linguistic content and emotional valence on listeners’ ratings of speech stimuli.

Comparing speech to non-speech, our results revealed that listeners judged strength comparably for distressed speech and screams, but were more sensitive to variation in strength, and estimated strength more reliably, from roars than from aggressive speech (see *γ* (sensitivity) and *R*^*2*^ (reliability) in statistical analyses). Thus, roars communicated strength more reliably than aggressive speech, but also *exaggerated* strength more effectively. These results accord with evidence that affective information is preferentially decoded from nonverbal vocalizations over emotionally inflected speech [83,84], suggesting that nonverbal vocalizations may, in certain contexts, be more effective carriers of motivational and indexical cues than speech. Interestingly, recent work has further shown that identity-related information is more effectively encoded in volitional than in spontaneous laughter [27].

Our results build on evidence by Sell and colleagues that listeners can accurately assess strength from neutral speech stimuli [11], showing here that listeners can also detect strength from emotional speech and nonverbal vocalizations. However, with the exception of female distressed speech, this ability was limited only to aggressive stimuli. Thus, aggressively motivated vocal behavior, whether in the form of speech or nonverbal vocalizations, appears to be optimised to communicate threat potential. These results are consistent with an extensive body of research demonstrating that listeners attend to formidability cues in aggressive calls across a wide range of mammals (e.g., giant pandas [2], sea lions [3], fallow and red deer [4,5], and dogs [6]). Moreover, the fact that variation in strength was generally not detected in distress stimuli indicates that the availability of formidability cues varies with the putative function of the signal, possibly reflecting differential selection on vocalizers to encode formidability cues in aggressive rather than submissive voice signals.

Listeners were less sensitive to variation in actual height than strength, and estimated height less reliably. Nevertheless, they could detect a small but significant proportion of variation in height from male and female distressed speech, female aggressive speech, and male distress screams. Compared to other stimulus types, these stimuli were on average characterized by relatively lower F0, thus facilitating formant perception through increased spectral density [8,85]. They were also characterized by less amplitude modulation than were other stimulus types, thus minimising the interference of sidebands with formant perception. Listeners may have therefore utilised formant cues to estimate height from these vocal stimuli. Our results are consistent with previous work indicating that listeners are only moderately accurate in voice-based estimates of body size for natural height distributions and on the basis of neutral speech stimuli, such as vowel sounds [8–10].

The finding that F0 predicted listeners’ height ratings but not actual height suggests that F0 may have confounded accurate height assessment. Many studies report a consistent perceptual bias in listeners to associate low-F0 speech with larger body size at the within-sex level [8–10,86–90], despite F0 being a very poor predictor of body size when controlling for sex and age [58]. We show that this bias, potentially driven by overgeneralization of sound-size relationships [9] and long thought to interfere with accurate body size estimation ([91,92,9] but see [8]), extends beyond speech to judgments of nonverbal vocalizations. While it has also been reported that low F0 may elicit higher strength attributions in neutral speech [11], our study did not corroborate this finding.

As strength and height were not correlated in the present study, our results provide strong evidence that the human voice contains independent cues to strength and height and that strength cues may be more perceptually salient. This finding complements the greater relevance of physical strength than body size to perceptions of men’s fighting ability [51] and bodily attractiveness [93] from images, where absolute strength may be easier to gauge from individual images of bodies than absolute size.

Contrary to some previous studies, we did not find evidence that strength and height are more reliably estimated from male than female voices [9,11], nor that male listeners are more sensitive than female listeners to acoustic cues to body size (e.g., [7] but see [9]). Thus, accuracy in strength and size estimation was largely unaffected by the sex of the vocalizer or listener. Yet male vocalizers were, in reality, both physically stronger and larger than were female vocalizers due to sexual dimorphism in the human body. Listeners’ estimates of height correctly reflected this dimorphism in body size, as males were consistently judged as taller than females (though particularly for aggressive and nonverbal vocalisations). In contrast, listeners did not consistently rate male vocalizers as stronger than females. Rather, males were only rated as stronger than females by male listeners, and only for judgments of aggressive roars.

These sex effects partly corroborate those reported in a recent study on relative voice-based judgments of strength and body size [12]. In that study, where we utilized the same roars and aggressive speech sentences as those used here, listeners were more likely to judge vocalizers as taller and stronger relative to themselves when those vocalizers produced roars compared to aggressive speech. This ‘exaggerating effect’ of roaring only worked for male vocalizers. Moreover, male listeners generally underestimated the size and strength of female vocalizers relative to their own, whereas female listeners overestimated the size and strength of male vocalizers. While the results of the present study are not immediately comparable due to differences in the nature of the task (i.e., absolute versus relative judgments of strength and size), an interesting pattern emerging in both studies is that roars appear to exaggerate strength and size, particularly for men.

In the playback experiments presented here, listeners’ ratings of strength and height were absolute and given on a scale (“Rate how strong/tall this vocalizer is”), similar to the method used by Sell and colleagues [11], thus facilitating cross-study comparisons. Other studies have asked listeners to judge the absolute height of vocalizers in centimetres (e.g., [91]) or the relative height of two same-sex vocalizers [8,9,89]. More recently [12], listeners were tasked for the first time with judging the strength and size of vocalizers *relative to their own*. While the results of these varied studies indicate that listeners can judge strength and size from the voice using either absolute or relative scales, listeners appear particularly accurate when judging the strength and size of others relative to themselves, perhaps because such a task seems the most ecologically valid and thus easiest (12). We recommend that researchers now examine the acoustic correlates of listeners’ relative strength judgments, as this could reveal more consistent and robust effects.

Finally, in the present study, male and female voices were presented in separate blocks. While it is possible that such a design could encourage listeners to judge the strength or size of vocalizers relative to others of the same rather than opposite sex, listeners consistently judged males as larger than females despite a similar blocking design, suggesting that blocking by sex did not substantially influence listeners’ ratings.

## General discussion

We compared the acoustic structure of aggressive roars, distress screams, and their valenced speech equivalents (Experiment 1), and examined the effectiveness of these various speech stimuli in communicating physical strength (Experiment 2) and height (Experiment 3) to listeners. Our results provide strong evidence that the acoustic structure of human aggressive and distress vocal signals, particularly nonverbal vocalizations (roars and screams), varies according to Morton’s motivational-structural rules [30]. Accordingly, aggressive stimuli exaggerated impressions of strength and body size relative to distress stimuli. Corroborating previous attempts [11,15,16], our acoustic analyses did not identify vocal features that reliably mediated the communication of strength, yet listeners could nevertheless accurately estimate strength from male and female aggressive (but not distress) vocal stimuli, and most reliably from aggressive roars. To a lesser degree, listeners could also estimate the height of vocalizers. Roars therefore conveyed honest inter-individual variation in strength more reliably than did any other type of vocal stimulus, and also exaggerated impressions of physical formidability most effectively.

The acoustic basis by which physical formidability (particularly strength) is communicated therefore remains unclear. Loudness and roughness were consistently associated with higher strength ratings, whereas loudness and lower F0 were often associated with higher height ratings, but these acoustic characteristics did not predict actual strength or height, and thus cannot account for the ability of listeners to reliably estimate strength, and to a lesser degree, height, solely from the acoustic structure of vocal stimuli. Similarly, while listeners detected strength variation in voice conditions for which the dominant formant frequency (DFF4) negatively correlated with actual strength, DFF4 did not predict listeners’ strength ratings. Listeners also detected strength variation from male aggressive speech and roars despite the absence of acoustic predictors of actual strength for these stimuli. Thus, despite measuring a wide set of relevant acoustic characteristics, our analyses failed to determine the acoustic pathways that mediate strength communication, confirming previous observations based on fewer vocal parameters–namely F0 and formants [11,15–17].

Despite a lack of robust vocal indices of actual physical formidability, this research provides compelling evidence that volitional voice production in an aggressive or submissive context effectively and respectively maximizes or minimizes listeners’ impressions of a vocalizer’s strength and body size (see also [29]). Differences in the acoustic structure of aggressive and distressed vocal stimuli support the exploitation of perceptual biases linking low and harsh voice frequencies to large body size and dominance [8,9,30,31,33,90,94]. Further experimental research is now needed to elucidate the relative roles of emotional context (aggression versus distress) and vocal stimulus type (nonverbal vocalisation versus speech) on listeners’ strength ratings, as both variables accounted for variance in the accuracy of listeners’ judgments.

The vocal stimuli used in this study were collected through acted scenarios and hence our results provide novel insight into both the acoustic structure, and probable social functions, of voice modulation and deception. Indeed, the ability to exaggerate one’s size or strength through vocal production is likely to have conferred an evolutionary advantage, as both larger body size and greater strength are associated with various socioeconomic, competitive, and mating benefits [93,95–100]. In line with our findings, other recent evidence indicates that the capacity to volitionally exaggerate or minimize body size via simulated nonverbal emotional expressions is not limited to actors [101,102]. In our study, screams and roars, while volitionally produced, nevertheless had the largest effect on listeners’ ratings of strength and height. This, paired with recent work showing that listeners can effectively estimate pain intensity from simulated pain cries [29], is consistent with the emerging hypothesis that deceptive voice modulation may be at the origins of selection for humans’ uniquely advanced vocal control abilities [20,65,103]. Indeed, some nonhuman mammals already demonstrate a limited capacity for functional vocal deception [103] and body size exaggeration [75,77,47,20] in agonistic contexts, as well as more voluntary vocal flexibility recently observed in nonhuman primates ([104–106] see also [20] for review). Survival benefits conferred to those able to modulate the expression of primary indexical cues may have given rise to increasingly greater vocal control, paving the way for the evolution of complex speech capabilities [20,103].

However, while the co-optation of primary relationships between acoustic cues and physical attributes may more effectively serve motivational signalling, variation in individuals’ capacity to modulate these cues may result in a decoupling between the cues and attributes. This may partly account for the lack of consistent acoustic correlates of actual height or strength observed here and in previous work. Interestingly, that listeners were able to accurately gauge strength from simulated roars and screams suggests that they could detect vocal deception and adjust their judgments accordingly. Evolutionary accounts of vocal signalling contend that, in agonistic or competitive contexts, vocalizers should evolve strategies to better manipulate receivers (thus obfuscating indexical information in favour of motivational signalling), while receivers should evolve ways to detect and resist such manipulation (thus reliably estimating indexical characteristics in spite of deceptive voice modulation) [103,107,108]. In future work, acoustic analyses could be used to investigate whether cues to deception are encoded in nonverbal vocalizations (e.g., whether roars elicited in natural versus simulated contexts vary structurally), and playback experiments could be employed to assess whether listeners can differentiate between natural and simulated vocalisations, or detect volitional vocal exaggeration or minimisation of traits such as body size and strength. Researchers may also examine whether other nonverbal vocalizations relevant to the signalling of formidability (e.g. martial arts kiaps) communicate indexical cues, and whether these vocalizations more reliably communicate motivational state than does speech (e.g. aggression, submission, distress, experienced pain).

It is possible that cues to strength and body size were communicated by acoustic characteristics that were not captured by our acoustic analyses. For example, information may be contained in the dynamic temporal variation of these vocal parameters; indeed, such information is commonly utilised in the construction of model-based emotion recognition from speech [109–111]. Listeners may also rely on complex linear or nonlinear combinations of acoustic parameters. While analysis of the individual contribution of acoustic characteristics has revealed numerous indexical cues in human and nonhuman mammal vocal behavior [112], future research should utilise alternative acoustic analytical approaches (e.g. linear interactions between acoustic characteristics, deep neural networks, hidden Markov models) to elucidate more complex acoustic mechanisms potentially communicating not only inter-individual variation in strength, but also other functional cues which linear acoustic analysis has been unable to account for (e.g., sex discrimination from babies’ cries [55]).

### Conclusion

We show that listeners can detect variation in vocalizer strength and body size from simulated nonverbal and verbal vocal stimuli produced in agonistic contexts (aggression and distress, i.e., contexts in which the communication of physical formidability is most ecologically relevant). Roars were particularly effective in communicating strength; the lack of linguistic constraints on aggressive roars appears to afford a greater acoustic space with which to both honestly communicate variation in strength between individuals, and exaggerate strength relative to other vocal signals within individuals. These results complement studies examining the vocal communication and exaggeration of physical traits and threat in nonhuman mammal species [5,44,45,47,78] and add to a growing body of evidence indicating structural and functional homology between human and nonhuman mammal vocalizations such as laughter [18–21] and infant distress cries [22–24]. Nonverbal vocalizations, and the ability to voluntary produce and modulate them, may constitute a direct intermediary link between involuntary control of stereotyped calls in nonhuman mammals, and full-blown volitional speech in humans [20,65,103]. As such, further investigation into the structure and function of nonverbal vocalizations may be essential to understanding the origins and evolution of human vocal communication (both verbal and nonverbal), and its relationship to animal vocal signals.

## Supporting information

S1 Tables Supplementary tables. (DOCX)

S1 Text Full protocol of acoustic analysis. (DOCX)

S2 Text Significant zero-order correlations. (DOCX)

S1 File Dataset. (XLSX)

## References

[1] Andersson MB. Sexual selection. Princeton, NJ: Princeton University Press; 1994.

[2] Charlton BD, Zhihe Z, Snyder RJ. Giant pandas perceive and attend to formant frequency variation in male bleats. Anim Behav. 2010;79: 1221–1227. 10.1016/j.anbehav.2010.02.018

[3] Charrier I, Ahonen H, Harcourt RG. What makes an Australian sea lion (Neophoca cinerea) male’s bark threatening? J Comp Psychol. 2011;125: 385–392. 10.1037/a0024513 21767004

[4] Pitcher BJ, Briefer EF, McElligott AG. Intrasexual selection drives sensitivity to pitch, formants and duration in the competitive calls of fallow bucks. BMC Evol Biol. 2015;15: 149 10.1186/s12862-015-0429-7 26279584 PMC4538740

[5] Reby D, McComb K, Cargnelutti B, Darwin C, Fitch WT, Clutton-Brock T. Red deer stags use formants as assessment cues during intrasexual agonistic interactions. Proc R Soc Lond B Biol Sci. 2005;272: 941–947. 10.1098/rspb.2004.2954 16024350 PMC1564087

[6] Taylor AM, Reby D, McComb K. Size communication in domestic dog, Canis familiaris, growls. Anim Behav. 2010;79: 205–210. 10.1016/j.anbehav.2009.10.030

[7] Charlton BD, Taylor AM, Reby D. Are men better than women at acoustic size judgements? Biol Lett. 2013;9: 20130270 10.1098/rsbl.2013.0270 23720522 PMC3730639

[8] Pisanski K, Fraccaro PJ, Tigue CC, O’Connor JJM, Feinberg DR. Return to Oz: Voice pitch facilitates assessments of men’s body size. J Exp Psychol Hum Percept Perform. 2014;40: 1316–1331. 10.1037/a0036956 24933617

[9] Rendall D, Vokey JR, Nemeth C. Lifting the curtain on the Wizard of Oz: Biased voice-based impressions of speaker size. J Exp Psychol Hum Percept Perform. 2007;33: 1208–1219. 10.1037/0096-1523.33.5.1208 17924818

[10] Pisanski K, Feinberg D, Oleszkiewicz A, Sorokowska A. Voice cues are used in a similar way by blind and sighted adults when assessing women’s body size. Sci Rep. 2017;7: 10329 10.1038/s41598-017-10470-3 28871192 PMC5583321

[11] Sell A, Bryant GA, Cosmides L, Tooby J, Sznycer D, von Rueden C, et al Adaptations in humans for assessing physical strength from the voice. Proc R Soc B Biol Sci. 2010;277: 3509–3518. 10.1098/rspb.2010.0769 20554544 PMC2982226

[12] Raine J, Pisanski K, Oleszkiewicz A, Simner J, Reby D. Human listeners can accurately judge relative strength and height from aggressive roars and speech. iScience. 2018;4: 273–280. 10.1016/j.isci.2018.05.002 30240746 PMC6146593

[13] Hodges-Simeon CR, Gurven M, Puts DA, Gaulin SJC. Vocal fundamental and formant frequencies are honest signals of threat potential in peripubertal males. Behav Ecol. 2014; aru081 10.1093/beheco/aru081 25024638 PMC4095947

[14] Puts DA, Apicella CL, Cárdenas RA. Masculine voices signal men’s threat potential in forager and industrial societies. Proc R Soc Lond B Biol Sci. 2012;279: 601–609. 10.1098/rspb.2011.0829 21752821 PMC3234546

[15] Smith KM, Olkhov YM, Puts DA, Apicella CL. Hadza men with lower voice pitch have a better hunting reputation. Evol Psychol. 2017;15: 1474704917740466 10.1177/1474704917740466 29179581 PMC10481060

[16] Han C, Wang H, Fasolt V, Hahn AC, Holzleitner IJ, Lao J, et al No clear evidence for correlations between handgrip strength and sexually dimorphic acoustic properties of voices. Am J Hum Biol. 2018;30: e23178 10.1002/ajhb.23178 30251293

[17] Kordsmeyer TL, Hunt J, Puts DA, Ostner J, Penke L. The relative importance of intra-and intersexual selection on human male sexually dimorphic traits. Evol Hum Behav. 2018;

[18] Davila-Ross M, Owren MJ, Zimmermann E. Reconstructing the evolution of laughter in great apes and humans. Curr Biol. 2009;19: 1106–1111. 10.1016/j.cub.2009.05.028 19500987

[19] Davila-Ross M, Owren MJ, Zimmermann E. The evolution of laughter in great apes and humans. Commun Integr Biol. 2010;3: 191–194. 10.4161/cib.3.2.10944 20585520 PMC2889984

[20] Pisanski K, Cartei V, McGettigan C, Raine J, Reby D. Voice modulation: A window into the origins of human vocal control? Trends Cogn Sci. 2016;20: 304–318. 10.1016/j.tics.2016.01.002 26857619

[21] Bryant GA, Aktipis CA. The animal nature of spontaneous human laughter. Evol Hum Behav. 2014;35: 327–335. 10.1016/j.evolhumbehav.2014.03.003

[22] Lingle S, Riede T. Deer mothers are sensitive to infant distress vocalizations of diverse mammalian species. Am Nat. 2014;184: 510–522. 10.1086/677677 25226186

[23] Lingle S, Wyman MT, Kotrba R, Teichroeb LJ, Romanow CA. What makes a cry a cry? A review of infant distress vocalizations. Curr Zool. 2012;58: 698–726.

[24] Zeifman DM. An ethological analysis of human infant crying: Answering Tinbergen’s four questions. Dev Psychobiol. 2001;39: 265–285. 10.1002/dev.1005 11745323

[25] Bryant GA, Fessler DMT, Fusaroli R, Clint E, Aarøe L, Apicella CL, et al Detecting affiliation in colaughter across 24 societies. Proc Natl Acad Sci. 2016;113: 4682–4687. 10.1073/pnas.1524993113 27071114 PMC4855576

[26] Scott SK, Lavan N, Chen S, McGettigan C. The social life of laughter. Trends Cogn Sci. 2014;18: 618–620. 10.1016/j.tics.2014.09.002 25439499 PMC4255480

[27] Lavan N, Short B, Wilding A, McGettigan C. Impoverished encoding of speaker identity in spontaneous laughter. Evol Hum Behav. 2018;39: 139–145.

[28] Raine J, Pisanski K, Reby D. Tennis grunts communicate acoustic cues to sex and contest outcome. Anim Behav. 2017;130: 47–55.

[29] Raine J, Pisanski K, Simner J, Reby D. Vocal communication of simulated pain. Bioacoustics. 2018; 1–23. 10.1080/09524622.2018.1463295

[30] Morton ES. On the occurrence and significance of motivation-structural rules in some bird and mammal sounds. Am Nat. 1977;111: 855–869.

[31] Ohala JJ. An ethological perspective on common cross-language utilization of F0 of voice. Phonetica. 1984;41: 1–16. 10.1159/000261706 6204347

[32] Owren MJ, Rendall D. Sound on the rebound: Bringing form and function back to the forefront in understanding nonhuman primate vocal signaling. Evol Anthropol Issues News Rev. 2001;10: 58–71. 10.1002/evan.1014

[33] Owings DH, Morton ES. Animal vocal communication: A new approach. Cambridge University Press; 1998.

[34] Bernstein IS, Ehardt CL. Agonistic aiding: Kinship, rank, age, and sex influences. Am J Primatol. 1985;8: 37–52. 10.1002/ajp.1350080105 31986822

[35] Hogstedt G. Adaptation unto death: function of fear screams. Am Nat. 1983;121: 562–570. 10.1086/284083

[36] Slocombe KE, Zuberbühler K. Chimpanzees modify recruitment screams as a function of audience composition. Proc Natl Acad Sci. 2007;104: 17228–17233. 10.1073/pnas.0706741104 17942683 PMC2040427

[37] Conlan T. The nature of warfare in fourteenth-century Japan: The record of Nomoto Tomoyuki. J Jpn Stud. 1999;25: 299–330. 10.2307/133314

[38] Merridale C. Culture, ideology and combat in the Red Army, 1939–45. J Contemp Hist. 2006;41: 305–324.

[39] Rance P. War Cry. The encyclopedia of the Roman army. John Wiley & Sons, Ltd; 2015 10.1002/9781118318140.wbra1621

[40] Blanchard CD, Hynd AL, Minke KA, Minemoto T, Blanchard RJ. Human defensive behaviors to threat scenarios show parallels to fear- and anxiety-related defense patterns of non-human mammals. Neurosci Biobehav Rev. 2001;25: 761–770. 10.1016/S0149-7634(01)00056-2 11801300

[41] Kim J, Heshka S, Gallagher D, Kotler DP, Mayer L, Albu J, et al Intermuscular adipose tissue-free skeletal muscle mass: estimation by dual-energy X-ray absorptiometry in adults. J Appl Physiol. 2004;97: 655–660. 10.1152/japplphysiol.00260.2004 15090482

[42] Shen W, Punyanitya M, Wang Z, Gallagher D, St.-Onge M-P, Albu J, et al Total body skeletal muscle and adipose tissue volumes: estimation from a single abdominal cross-sectional image. J Appl Physiol. 2004;97: 2333–2338. 10.1152/japplphysiol.00744.2004 15310748

[43] Stoll T, Huber E, Seifert B, Michel BA, Stucki G. Maximal isometric muscle strength: Normative values and gender-specific relation to age. Clin Rheumatol. 2000;19: 105–113. 10.1007/s100670050026 10791620

[44] Charlton BD, Ellis WAH, McKinnon AJ, Cowin GJ, Brumm J, Nilsson K, et al Cues to body size in the formant spacing of male koala (Phascolarctos cinereus) bellows: honesty in an exaggerated trait. J Exp Biol. 2011;214: 3414–3422. 10.1242/jeb.061358 21957105

[45] Fitch WT, Hauser MD. Unpacking “honesty”: Vertebrate vocal production and the evolution of acoustic signals In: Simmons AM, Fay RR, Popper AN, editors. Acoustic Communication. Springer New York; 2003 pp. 65–137. Available: http://link.springer.com/chapter/10.1007/0-387-22762-8_3

[46] Fitch WT, Reby D. The descended larynx is not uniquely human. Proc R Soc Lond B Biol Sci. 2001;268: 1669–1675. 10.1098/rspb.2001.1704 11506679 PMC1088793

[47] Reby D, McComb K. Anatomical constraints generate honesty: acoustic cues to age and weight in the roars of red deer stags. Anim Behav. 2003;65: 519–530. 10.1006/anbe.2003.2078

[48] Titze IR. Principles of voice production. National Center for Voice and Speech; 1994.

[49] Wagner WE Jr. Deceptive or honest signalling of fighting ability? A test of alternative hypotheses for the function of changes in call dominant frequency by male cricket frogs. Anim Behav. 1992;44, Part 3: 449–462. 10.1016/0003-3472(92)90055-E

[50] Zahavi A, Zahavi A. The handicap principle: A missing piece of Darwin’s puzzle. Oxford: Oxford University Press; 1997.

[51] Sell A, Cosmides L, Tooby J, Sznycer D, Rueden C von, Gurven M. Human adaptations for the visual assessment of strength and fighting ability from the body and face. Proc R Soc Lond B Biol Sci. 2009;276: 575–584. 10.1098/rspb.2008.1177 18945661 PMC2664345

[52] Boersma P, Weenink D. Praat: doing phonetics by computer [Internet]. 2017 Available: http://www.praat.org/

[53] Kreiman J, Sidtis D. Foundations of voice studies: An interdisciplinary approach to voice production and perception [Internet]. Wiley-Blackwell; 2011 Available: http://eu.wiley.com/WileyCDA/WileyTitle/productCd-0631222979.html

[54] Koutseff A, Reby D, Martin O, Levrero F, Patural H, Mathevon N. The acoustic space of pain: cries as indicators of distress recovering dynamics in pre-verbal infants. Bioacoustics. 2017;0: 1–13. 10.1080/09524622.2017.1344931

[55] Reby D, Levréro F, Gustafsson E, Mathevon N. Sex stereotypes influence adults’ perception of babies’ cries. BMC Psychol. 2016;4 10.1186/s40359-016-0123-6 27079192 PMC4832517

[56] Paliwal KK. Spectral subband centroid features for speech recognition. Proceedings of the 1998 IEEE International Conference on Acoustics, Speech and Signal Processing. 1998. pp. 617–620. 10.1109/ICASSP.1998.675340

[57] Rendall D, Kollias S, Ney C, Lloyd P. Pitch (F0) and formant profiles of human vowels and vowel-like baboon grunts: the role of vocalizer body size and voice-acoustic allometry. J Acoust Soc Am. 2005;117: 944–955. 15759713 10.1121/1.1848011

[58] Pisanski K, Fraccaro PJ, Tigue CC, O’Connor JJM, Röder S, Andrews PW, et al Vocal indicators of body size in men and women: a meta-analysis. Anim Behav. 2014;95: 89–99. 10.1016/j.anbehav.2014.06.011

[59] Mundry R, Sommer C. Discriminant function analysis with nonindependent data: consequences and an alternative. Anim Behav. 2007;74: 965–976. 10.1016/j.anbehav.2006.12.028

[60] Sauter DA, Eisner F, Calder AJ, Scott SK. Perceptual cues in nonverbal vocal expressions of emotion. Q J Exp Psychol. 2010;63: 2251–2272. 10.1080/17470211003721642 20437296 PMC4178283

[61] Bachorowski JA, Smoski MJ, Owren MJ. The acoustic features of human laughter. J Acoust Soc Am. 2001;110: 1581–1597. 10.1121/1.1391244 11572368

[62] Szameitat DP, Darwin CJ, Szameitat AJ, Wildgruber D, Alter K. Formant characteristics of human laughter. J Voice. 2011;25: 32–37. 10.1016/j.jvoice.2009.06.010 20381307

[63] Scott SK, Sauter D, McGettigan C. Brain mechanisms for processing perceived emotional vocalizations in humans. Handb Behav Neurosci. 2010;19: 187–197. 10.1016/B978-0-12-374593-4.00019-X

[64] Titze IR. Human speech: A restricted use of the mammalian larynx. J Voice. 2017;31: 135–141. 10.1016/j.jvoice.2016.06.003 27397113 PMC5219873

[65] Brown S. A joint prosodic origin of language and music. Front Psychol. 2017;8 10.3389/fpsyg.2017.01894 29163276 PMC5666296

[66] Miller SE, Schlauch RS, Watson PJ. The effects of fundamental frequency contour manipulations on speech intelligibility in background noise. J Acoust Soc Am. 2010;128: 435–443. 10.1121/1.3397384 20649237

[67] Traunmüller H, Eriksson A. Acoustic effects of variation in vocal effort by men, women, and children. J Acoust Soc Am. 2000;107: 3438–51. 10875388 10.1121/1.429414

[68] Behrman A. Speech and voice science. San Diego, California: Plural Pub; 2007.

[69] Herbst CT. Biophysics of vocal production in mammals Vertebrate sound production and acoustic communication. Springer, Cham; 2016 pp. 159–189. 10.1007/978-3-319-27721-9_6

[70] Berry DA, Herzel H, Titze IR, Story BH. Bifurcations in excised larynx experiments. J Voice. 1996;10: 129–138. 10.1016/S0892-1997(96)80039-7 8734387

[71] Fitch WT, Neubauer J, Herzel H. Calls out of chaos: the adaptive significance of nonlinear phenomena in mammalian vocal production. Anim Behav. 2002;63: 407–418. 10.1006/anbe.2001.1912

[72] Jiang JJ, Zhang Y, Stern J. Modeling of chaotic vibrations in symmetric vocal folds. J Acoust Soc Am. 2001;110: 2120–2128. 10.1121/1.1395596 11681389

[73] Zhang Y, Jiang JJ. Spatiotemporal chaos in excised larynx vibrations. Phys Rev E. 2005;72: 035201 10.1103/PhysRevE.72.035201 16241503

[74] Gouzoules H, Gouzoules S. Agonistic screams differ among four species of macaques: the significance of motivation-structural rules. Anim Behav. 2000;59: 501–512. 10.1006/anbe.1999.1318 10715171

[75] de Boer B, Wich SA, Hardus ME, Lameira AR. Acoustic models of orangutan hand-assisted alarm calls. J Exp Biol. 2015;218: 907–914. 10.1242/jeb.110577 25788727

[76] Fox MW. A comparative study of the development of facial expressions in canids; wolf, coyote and foxes. Behaviour. 1970;36: 49–73.

[77] Hardus ME, Lameira AR, Schaik CPV, Wich SA. Tool use in wild orang-utans modifies sound production: a functionally deceptive innovation? Proc R Soc Lond B Biol Sci. 2009; rspb20091027 10.1098/rspb.2009.1027 19656794 PMC2817314

[78] Harris TR, Fitch WT, Goldstein LM, Fashing PJ. Black and white colobus monkey (Colobus guereza) roars as a source of both honest and exaggerated information about body mass. Ethology. 2006;112: 911–920.

[79] Hill AK, Bailey DH, Puts DA. Gorillas in our midst? Human sexual dimorphism and contest competition in men In: Tibayrenc M, Ayala FJ, editors. On Human Nature. San Diego: Academic Press; 2017 pp. 235–249. 10.1016/B978-0-12-420190-3.00015-6

[80] Hill AK, Hunt J, Welling LLM, Cárdenas RA, Rotella MA, Wheatley JR, et al Quantifying the strength and form of sexual selection on men’s traits. Evol Hum Behav. 2013;34: 334–341. 10.1016/j.evolhumbehav.2013.05.004

[81] Chandler J, Shapiro D. Conducting clinical research using crowdsourced convenience samples. Annu Rev Clin Psychol. 2016;12: 53–81. 10.1146/annurev-clinpsy-021815-093623 26772208

[82] Hughes JEA, Gruffydd E, Simner J, Ward J. Synaesthesia aids in savant-skill acquisition: Training calendar calculation in sequence-space synaesthetes. Cortex. in press; 10.1016/j.cortex.2018.11.023 30605870

[83] Pell MD, Rothermich K, Liu P, Paulmann S, Sethi S, Rigoulot S. Preferential decoding of emotion from human non-linguistic vocalizations versus speech prosody. Biol Psychol. 2015;111: 14–25. 10.1016/j.biopsycho.2015.08.008 26307467

[84] Scott SK, Young AW, Calder AJ, Hellawell DJ, Aggleton JP, Johnsons M. Impaired auditory recognition of fear and anger following bilateral amygdala lesions. Nature. 1997;385: 254–257. 10.1038/385254a0 9000073

[85] Ryalls JH, Lieberman P. Fundamental frequency and vowel perception. J Acoust Soc Am. 1982;72: 1631–1634. 10.1121/1.388499 7175033

[86] Collins SA. Men’s voices and women’s choices. Anim Behav. 2000;60: 773–780. 10.1006/anbe.2000.1523 11124875

[87] Feinberg DR, Jones BC, Little AC, Burt DM, Perrett DI. Manipulations of fundamental and formant frequencies influence the attractiveness of human male voices. Anim Behav. 2005;69: 561–568. 10.1016/j.anbehav.2004.06.012

[88] Pisanski K, Isenstein SGE, Montano KJ, O’Connor JJM, Feinberg DR. Low is large: spatial location and pitch interact in voice-based body size estimation. Atten Percept Psychophys. 2017;79: 1239–1251. 10.3758/s13414-016-1273-6 28229428

[89] Pisanski K, Rendall D. The prioritization of voice fundamental frequency or formants in listeners’ assessments of speaker size, masculinity, and attractiveness. J Acoust Soc Am. 2011;129: 2201–2212. 10.1121/1.3552866 21476675

[90] Smith DR, Patterson RD. The interaction of glottal-pulse rate and vocal-tract length in judgements of speaker size, sex, and age. J Acoust Soc Am. 2005;118: 3177–3186. 16334696 10.1121/1.2047107 PMC2346770

[91] Bruckert L, Liénard J-S, Lacroix A, Kreutzer M, Leboucher G. Women use voice parameters to assess men’s characteristics. Proc R Soc Lond B Biol Sci. 2006;273: 83–89. 10.1098/rspb.2005.3265 16519239 PMC1560007

[92] Greisbach R. Estimation of speaker height from formant frequencies. Int J Speech Lang Law. 1999;6: 265–277.

[93] Sell A, Lukazsweski AW, Townsley M. Cues of upper body strength account for most of the variance in men’s bodily attractiveness. Proc R Soc B. 2017;284: 20171819 10.1098/rspb.2017.1819 29237852 PMC5745404

[94] Puts DA, Hodges CR, Cárdenas RA, Gaulin SJC. Men’s voices as dominance signals: vocal fundamental and formant frequencies influence dominance attributions among men. Evol Hum Behav. 2007;28: 340–344. 10.1016/j.evolhumbehav.2007.05.002

[95] Brues A. The spearman and the archer—an essay on selection in body build. Am Anthropol. 1959;61: 457–469. 10.1525/aa.1959.61.3.02a00080

[96] Frederick DA, Haselton MG. Why is muscularity sexy? Tests of the fitness indicator hypothesis. Pers Soc Psychol Bull. 2007;33: 1167–1183. 10.1177/0146167207303022 17578932

[97] Gallup AC, White DD, Gallup GG. Handgrip strength predicts sexual behavior, body morphology, and aggression in male college students. Evol Hum Behav. 2007;28: 423–429. 10.1016/j.evolhumbehav.2007.07.001

[98] Judge TA, Cable DM. The effect of physical height on workplace success and income: Preliminary test of a theoretical model. J Appl Psychol. 2004;89: 428–441. 10.1037/0021-9010.89.3.428 15161403

[99] Monden CWS, Smits J. Maternal height and child mortality in 42 developing countries. Am J Hum Biol. 2009;21: 305–311. 10.1002/ajhb.20860 19107903

[100] Pisanski K, Feinberg DR. Cross-cultural variation in mate preferences for averageness, symmetry, body size, and masculinity. Cross-Cult Res. 2013;47: 162–197. 10.1177/1069397112471806

[101] Jürgens R, Grass A, Drolet M, Fischer J. Effect of acting experience on emotion expression and recognition in voice: Non-actors provide better stimuli than expected. J Nonverbal Behav. 2015;39: 195–214. 10.1007/s10919-015-0209-5 26246649 PMC4519627

[102] Pisanski K, Mora EC, Pisanski A, Reby D, Sorokowski P, Frackowiak T, et al Volitional exaggeration of body size through fundamental and formant frequency modulation in humans. Sci Rep. 2016;6 10.1038/srep34389 27687571 PMC5043380

[103] Oesch N. Deception as a derived function of language. Front Psychol. 2016;7 10.3389/fpsyg.2016.01485 27729895 PMC5037177

[104] Lameira AR, Hardus ME, Bartlett AM, Shumaker RW, Wich SA, Menken SBJ. Speech-like rhythm in a voiced and voiceless orangutan call. PLoS ONE. 2015;10: e116136 10.1371/journal.pone.0116136 25569211 PMC4287529

[105] Perlman M, Clark N. Learned vocal and breathing behavior in an enculturated gorilla. Anim Cogn. 2015;18: 1165–1179. 10.1007/s10071-015-0889-6 26139343

[106] Schel AM, Townsend SW, Machanda Z, Zuberbühler K, Slocombe KE. Chimpanzee alarm call production meets key criteria for intentionality. PLoS ONE. 2013;8: e76674 10.1371/journal.pone.0076674 24146908 PMC3797826

[107] Knight C. Ritual/speech coevolution: A solution to the problem of deception In: Hurford JR, Studdert-Kennedy M, Knight C, editors. Approaches to the evolution of language. Cambridge: Cambridge University Press; 1998 pp. 68–91.

[108] Krebs JR, Dawkins R. Animal signals: mind-reading and manipulation In: Krebs JR, Davies NB, editors. Behavioural ecology: An evolutionary approach. 2nd ed Oxford: Blackwell Scientific Publications; 1984 pp. 380–402.

[109] Le D, Provost EM. Emotion recognition from spontaneous speech using Hidden Markov models with deep belief networks. 2013 IEEE Workshop on Automatic Speech Recognition and Understanding. 2013. pp. 216–221. 10.1109/ASRU.2013.6707732

[110] Li L, Zhao Y, Jiang D, Zhang Y, Wang F, Gonzalez I, et al. Hybrid deep neural network–hidden Markov model (DNN-HMM) based speech emotion recognition. 2013 Humaine Association Conference on Affective Computing and Intelligent Interaction. 2013. pp. 312–317. 10.1109/ACII.2013.58

[111] Nwe TL, Foo SW, De Silva LC. Speech emotion recognition using hidden Markov models. Speech Commun. 2003;41: 603–623. 10.1016/S0167-6393(03)00099-2

[112] Taylor AM, Charlton BD, Reby D. Vocal production by terrestrial mammals: Source, filter, and function In: Suthers RA, Fitch WT, Fay RR, Popper AN, editors. Vertebrate Sound Production and Acoustic Communication. Springer International Publishing; 2016 pp. 229–259. 10.1007/978-3-319-27721-9_8

