# Supplementary material for: Human roars communicate upper-body strength more effectively than do screams or aggressive and distressed speech
Source: PLoS One. 2019 Mar 4;14(3):e0213034. doi: 10.1371/journal.pone.0213034 (PMC6398857; doi:10.1371/journal.pone.0213034)
Supplement: S1 Tables — (DOCX) [file pone.0213034.s001.docx]

**S1 Tables**

SUPPORTING INFORMATION

for:

Human roars communicate upper-body strength more effectively than do screams or aggressive and distressed speech

Jordan Raine, Katarzyna Pisanski, Rod Bond, Julia Simner, & David Reby

This Supporting Information document includes:

Supplementary Tables:

Table A. Factor loadings of acoustic variables on the discriminant functions (DF), for both sexes combined.

Table B. Factor loadings of acoustic variables on the discriminant functions (DF), for male vocalisers only.

Table C. Factor loadings of acoustic variables on the discriminant functions (DF), for female vocalisers only.

Table D. Standardised regression coefficients for acoustic predictors of men and women’s physical strength.

Table E. Standardised regression coefficients for acoustic predictors of men and women’s height.

Table F. Standardised regression coefficients for acoustic predictors of listeners’ ratings of men and women’s physical strength.

Table G. Standardised regression coefficients for acoustic predictors of listeners’ ratings of men and women’s height.

**Supplementary Tables**

|  | DF1 | DF2 | DF3 |
| --- | --- | --- | --- |
|  | Variance = 75% | Variance = 21% | Variance = 3% |
| Acoustic variable | Eigenvalue = 4.28 | Eigenvalue = 1.22 | Eigenvalue = 0.19 |
| Mean amplitude (dB) | **-.63** | -.03 | .34 |
| Intensity CV (dB) | **.51** | -.12 | .39 |
| Amplitude modulation (%) | **-.37** | -.03 | .06 |
| Major F0 inflections | **.20** | -.11 | -.14 |
| Minimum F0 (Hz) | -.27 | **.61** | .13 |
| Max F0 (Hz) | -.21 | **.57** | .30 |
| Mean F0 (Hz) | -.28 | **.57** | .33 |
| HNR (dB) | .06 | **.44** | .07 |
| Jitter (Hz) | -.19 | **-.24** | -.24 |
| Shimmer (dB) | -.03 | **.21** | -.21 |
| Dominant formant frequency DFF4 (Hz) | .03 | **.21** | -.01 |
| Start – end F0 (Hz) | -.02 | **-.20** | .02 |
| Time of max intensity (%) | .13 | .06 | **-.38** |
| Centre of gravity (Hz) | -.20 | .02 | **.33** |
| F0 CV (Hz) | .05 | -.01 | **.28** |
| Minor F0 inflections | .09 | .00 | **-.23** |

Table A. Factor loadings of acoustic variables on the discriminant functions (DF), for both sexes combined. Highest factor loadings (for each acoustic variable) are highlighted in bold.

Table B. Factor loadings of acoustic variables on the discriminant functions (DF), for male vocalisers only. Highest factor loadings (for each acoustic variable) are highlighted in bold.

|  | DF1 | DF2 | DF3 |
| --- | --- | --- | --- |
|  | Variance = 75% | Variance = 21% | Variance = 3% |
| Acoustic variable | Eigenvalue = 4.28 | Eigenvalue = 1.22 | Eigenvalue = 0.19 |
| Mean amplitude (dB) | **-.64** | -.06 | -.36 |
| Intensity CV (dB) | **.51** | -.04 | -.22 |
| Amplitude modulation (%) | **-.49** | -.23 | .13 |
| Major F0 inflections | **.18** | -.13 | .03 |
| Max F0 (Hz) | -.20 | **.62** | -.24 |
| Minimum F0 (Hz) | -.33 | **.54** | -.16 |
| Mean F0 (Hz) | -.31 | **.54** | -.28 |
| HNR (dB) | .13 | **.45** | .00 |
| Dominant formant frequency DFF4 (Hz) | .05 | **.25** | -.05 |
| Shimmer (dB) | -.05 | **.22** | .12 |
| Start – end F0 (Hz) | -.05 | **-.16** | .15 |
| Jitter (Hz) | -.24 | -.26 | **.33** |
| Centre of gravity (Hz) | -.26 | .00 | **-.30** |
| F0 CV (Hz) | .13 | .07 | **-.21** |
| Time of max intensity (%) | .14 | .02 | **.20** |
| Minor F0 inflections | .04 | -.09 | **.18** |

Table C. Factor loadings of acoustic variables on the discriminant functions (DF), for female vocalisers only. Highest factor loadings (for each acoustic variable) are highlighted in bold.

|  | DF1 | DF2 | DF3 |
| --- | --- | --- | --- |
|  | Variance = 75% | Variance = 21% | Variance = 3% |
| Acoustic variable | Eigenvalue = 4.28 | Eigenvalue = 1.22 | Eigenvalue = 0.19 |
| Mean amplitude (dB) | **.57** | -.03 | .26 |
| Intensity CV (dB) | **-.48** | -.14 | .43 |
| Amplitude modulation (%) | **.26** | .09 | .23 |
| Minimum F0 (Hz) | .21 | **.58** | .01 |
| Mean F0 (Hz) | .24 | **.49** | .22 |
| Max F0 (Hz) | .21 | **.45** | .19 |
| HNR (dB) | -.01 | **.40** | .05 |
| Jitter (Hz) | .14 | **-.21** | -.06 |
| Start – end F0 (Hz) | .00 | **-.20** | .18 |
| Dominant formant frequency DFF4 (Hz) | -.01 | **.15** | -.09 |
| Time of max intensity (%) | -.10 | .08 | **-.43** |
| Centre of gravity (Hz) | .13 | .03 | **.26** |
| F0 CV (Hz) | .01 | -.05 | **.24** |
| Shimmer (dB) | .02 | .18 | **-.23** |
| Minor F0 inflections | -.11 | .07 | **-.19** |
| Major F0 inflections | -.19 | -.06 | **-.19** |

Table D. Standardised regression coefficients for acoustic predictors of men and women’s physical strength. Separate stepwise regressions were computed for aggressive speech, aggressive roars, distressed speech, and distress screams.

| Acoustic Variable | Females | | | | Males | | | |
| --- | --- | --- | --- | --- | --- | --- | --- | --- |
|  | Agg S | Agg V | Dis S | Dis V | Agg S | Agg V | Dis S | Dis V |
| Duration (s) |  |  |  |  |  |  |  |  |
| Mean F0 (Hz) |  |  |  |  |  |  |  |  |
| Max F0 (Hz) |  |  |  |  |  |  |  |  |
| Min F0 (Hz) |  |  |  |  |  |  |  |  |
| Start – end F0 (Hz) |  |  |  |  |  |  |  |  |
| F0CV (Hz) |  |  |  |  |  |  |  |  |
| Minor F0 inflections |  |  |  |  |  |  |  |  |
| Major F0 inflections | **.42^*^** |  |  |  |  |  |  |  |
| Mean intensity (dB) |  |  |  |  |  |  |  |  |
| Time of max intensity (%) |  |  |  |  |  |  |  |  |
| intensity CV (dB) |  |  |  |  |  |  |  |  |
| Shimmer (dB) |  |  |  |  |  |  |  |  |
| Jitter (Hz) |  |  |  |  |  |  |  |  |
| HNR (dB) |  |  |  |  |  |  |  |  |
| Amplitude modulation (%) |  |  | **.55^**^** |  |  |  |  |  |
| Centre of gravity (Hz) |  |  |  |  |  |  |  |  |
| Dominant formant frequency DFF4 (Hz) | **-.47^**^** | **-.47^**^** | **-.32^*^** |  |  |  |  |  |

^*^ *p* < .05 ^**^ *p* < .01

| Acoustic Variable  Table E. Standardised regression coefficients for acoustic predictors of men and women’s height. Separate stepwise regressions were computed for aggressive speech, aggressive roars, distressed speech, and distress screams. | Females | | | | Males | | | |
| --- | --- | --- | --- | --- | --- | --- | --- | --- |
|  | Agg S | Agg V | Dis S | Dis V | Agg S | Agg V | Dis S | Dis V |
| Duration (s) |  |  |  |  |  |  |  |  |
| Mean F0 (Hz) |  |  |  |  |  |  |  |  |
| Max F0 (Hz) |  |  |  |  |  |  |  |  |
| Min F0 (Hz) | **-.39^*^** |  |  |  |  |  |  |  |
| Start – end F0 (Hz) |  |  |  |  |  |  |  |  |
| F0CV (Hz) |  |  |  |  | **-.47^**^** |  |  |  |
| Minor F0 inflections |  |  |  |  |  |  |  |  |
| Major F0 inflections |  |  |  |  |  |  |  |  |
| Mean intensity (dB) |  |  |  |  |  |  |  |  |
| Time of max intensity (%) |  |  |  |  |  |  |  |  |
| intensity CV (dB) |  | **.38^*^** |  |  |  |  |  |  |
| Shimmer (dB) |  |  |  |  |  |  |  |  |
| Jitter (Hz) |  |  |  |  |  |  |  |  |
| HNR (dB) |  |  |  |  |  |  |  |  |
| Amplitude modulation (%) |  |  | **.36^*^** |  |  |  |  |  |
| Centre of gravity (Hz) | **.55^**^** |  |  |  |  |  |  |  |
| Dominant formant frequency DFF4 (Hz) |  |  |  |  |  |  |  | **-.40^*^** |

^*^ *p* < .05 ^**^ *p* < .01

Table F. Standardised regression coefficients for acoustic predictors of listeners’ ratings of men and women’s physical strength. Separate stepwise regressions were computed for aggressive speech, aggressive roars, distressed speech, and distress screams.

| Acoustic Variable | Females | | | | Males | | | |
| --- | --- | --- | --- | --- | --- | --- | --- | --- |
|  | Agg S | Agg V | Dis S | Dis V | Agg S | Agg V | Dis S | Dis V |
| Duration (s) |  | **.53^***^** |  | **.21^*^** |  |  | **-.19^*^** |  |
| Mean F0 (Hz) |  |  |  |  |  |  |  | **-.49^***^** |
| Max F0 (Hz) |  |  | **-.29^***^** |  |  |  |  |  |
| Min F0 (Hz) |  |  |  |  |  |  |  |  |
| Start – end F0 (Hz) |  |  |  |  |  | **.40^**^** |  |  |
| F0CV (Hz) |  | **-.38^***^** |  |  |  |  | **-.28^**^** | **-.18^*^** |
| Minor F0 inflections |  |  |  |  |  |  |  |  |
| Major F0 inflections |  |  |  |  |  |  |  |  |
| Mean intensity (dB) | **.80^***^** | **.86^***^** | **1.11^***^** | **.71^***^** | **.37^**^** | **.36^*^** | **.95^***^** | **.60^***^** |
| Time of max intensity (%) |  |  |  |  |  |  |  |  |
| intensity CV (dB) |  |  |  |  |  | **-.34^*^** |  |  |
| Shimmer (dB) |  | **-.33^**^** |  |  | **-.18^*^** |  |  |  |
| Jitter (Hz) |  |  |  |  |  | **.43^**^** | **-.32^*^** |  |
| HNR (dB) | **-.35^***^** | **-.72^***^** | **-.34^***^** |  |  |  | **-.76^***^** | **-.23^*^** |
| Amplitude modulation (%) |  |  |  | **.40^***^** | **.58^***^** |  |  | **.40^**^** |
| Centre of gravity (Hz) |  |  |  |  |  |  |  |  |
| Dominant formant frequency DFF4 (Hz) |  |  |  |  |  |  |  |  |

^*^ *p* < .05 ^**^ *p* < .01 ^***^ *p* < .001

^+^ *p* < .10 ^*^ *p* < .05 ^**^ *p* < .01

| Acoustic Variable  Table G. Standardised regression coefficients for acoustic predictors of listeners’ ratings of men and women’s height. Separate stepwise regressions were computed for aggressive speech, aggressive roars, distressed speech, and distress screams. | Females | | | | Males | | | |
| --- | --- | --- | --- | --- | --- | --- | --- | --- |
|  | Agg S | Agg V | Dis S | Dis V | Agg S | Agg V | Dis S | Dis V |
| Duration (s) |  |  |  |  |  |  |  |  |
| Mean F0 (Hz) |  | **-.54^***^** |  |  | **-.49^*^** | **-.54^**^** | **-1.34^***^** | **-.80^***^** |
| Max F0 (Hz) |  |  | **-.34^*^** |  |  |  |  |  |
| Min F0 (Hz) |  |  |  |  |  |  | **.69^*^** |  |
| Start – end F0 (Hz) |  |  |  |  |  |  |  |  |
| F0CV (Hz) |  |  |  |  |  |  |  |  |
| Minor F0 inflections |  |  |  |  |  |  |  |  |
| Major F0 inflections |  |  |  |  |  |  |  |  |
| Mean intensity (dB) |  | **.70^***^** | **.78^***^** |  | **.76^**^** | **.32^*^** | **.90^***^** | **.46^**^** |
| Time of max intensity (%) |  |  |  |  |  | **-.28^*^** |  |  |
| intensity CV (dB) |  |  | **.32^**^** |  |  |  |  |  |
| Shimmer (dB) |  | **-.38^*^** |  |  |  |  |  |  |
| Jitter (Hz) |  |  |  |  |  | **.42^**^** |  | **.34^*^** |
| HNR (dB) |  |  | **-.45^***^** |  |  |  |  |  |
| Amplitude modulation (%) |  |  |  |  |  |  |  |  |
| Centre of gravity (Hz) | **.48^**^** |  |  | **-.52^**^** |  |  |  |  |
| Dominant formant frequency DFF4 (Hz) |  |  |  |  |  |  |  |  |

^*^ *p* < .05 ^**^ *p* < .01 ^***^ *p* < .001
