# Supplementary material for: Human roars communicate upper-body strength more effectively than do screams or aggressive and distressed speech
Source: PLoS One. 2019 Mar 4;14(3):e0213034. doi: 10.1371/journal.pone.0213034 (PMC6398857; doi:10.1371/journal.pone.0213034)
Supplement: S1 Text — (DOCX) [file pone.0213034.s002.docx]

**S1 Text**

SUPPORTING INFORMATION

for:

Human roars communicate upper-body strength more effectively than do screams or aggressive and distressed speech

Jordan Raine, Katarzyna Pisanski, Rod Bond, Julia Simner, David Reby

**S1 Text: Full protocol of acoustic analysis**

Vocal stimuli were analyzed using Praat 5.3.62 DSP package (Boersma & Weenink, 2017). Recordings were saved as WAV files at 44.1 kHz sampling frequency and 16 bits amplitude resolution.

We used a dedicated batch-processing script containing four distinct procedures. The F0 contour was first extracted using the *To Pitch (cc)* command. We systematically inspected each extracted pitch contour and verified it using a narrow band spectrogram displaying the first 2000 Hz of the signal. Erroneous pitch values (e.g., octave jumps) were manually corrected by selecting the appropriate F0 candidate values in the edited pitch object. In segments displaying subharmonics (where, in addition to F0, vocal fold vibration equal to an integer multiple of the fundamental frequency is present, Fitch, Neubauer, & Herzel, 2002), the F0 was systematically preferred over the subharmonic. For segments where deterministic chaos (aperiodic, irregular vocal fold vibration, Fitch et al., 2002) was present, the automatically extracted pitch contour generally did not select F0 values; where it did, we manually deselected these values. Each extracted F0 contour (pitch object) was saved as a text file for future reference. The F0 contour was used to derive the following parameters: mean F0, max F0, min F0, start-end F0 (a measure of the F0 contour), and F0CV (coefficient of variation of F0 over the duration of the signal). During inspection of each spectrogram, we also measured the proportion of the signal for which amplitude modulation was present, and created a measure representing this proportion as a percentage (%AM).

Next, two distinct smoothing algorithms (*Smooth…* command in Praat) were performed on the pitch contour: the first (*Smooth…* command parameter = 25), suppressed very short-term frequency fluctuation while preserving minor modulation events (such as frequency modulation), and the second (*Smooth*… command parameter = 2) only characterised major F0 modulation. Inflection points were counted (as each change in the sign of the contour’s derivative) after each smoothing procedure, and divided by the total duration of the voiced segments in each recording, resulting in two distinct indexes of F0 modulation (inflex25 - minor inflections, and inflex2 - major inflections).

A second procedure focused on the intensity contour and characterised the mean amplitude of the stimuli, the point at which the signal’s amplitude was highest (time of max intensity, expressed as a percentage of the signal’s duration), as well as amplitude variability by calculating intCV, the coefficient of variation of the intensity contour estimated using the *To intensity* command in Praat.

A third procedure focused on the periodic quality of the signal and measured harmonics-to-noise ratio (HNR, a measure of the ratio of harmonic spectral energy to chaotic spectral energy), jitter (small fluctuations in periodicity measured as the average of ‘local’, ‘rap’ and ‘ppq5’ measures in Praat) and shimmer (small variation in amplitude between consecutive periods, measured as the average of ‘local’, ‘apq5’ and ‘apq11’ parameters in Praat). While some researchers have argued that jitter and shimmer are largely inconsequential in the perception of non-pathological modal speech (Kreiman & Sidtis, 2011), these perturbation parameters may play a significant role in characterizing emotional nonverbal vocalizations. Indeed, acoustic analysis procedures similar to these have been applied successfully in previous studies of human babies’ cries (Koutseff et al., 2017; Reby, Levréro, Gustafsson, & Mathevon, 2016).

A final procedure characterized the spectral envelope of each vocal stimulus. Because many of the stimuli were relatively high-pitched (see Figure 1) and therefore characterised by a low spectral density, and because amplitude modulation (present in many stimuli, see Figure 1a, c) produces sidebands in frequency spectra that can be miscategorised as formants, formant frequencies were poorly defined and difficult to both perceive and measure via cepstrum or linear predictive coding analysis (Pisanski, Fraccaro, Tigue, O’Connor, & Feinberg, 2014; Ryalls & Lieberman, 1982). Instead, this procedure measured spectral centre of gravity (indicating where the ‘centre of mass’ of the spectrum is, calculated as the amplitude-weighted mean of the frequencies present in the signal), which also carries filter-related information (Paliwal, 1998).

Finally, as there is very little or no overlap in the distributions of the third and fourth formants (F3 and F4) across vowels (Abari, Rácz, & Olaszy, 2011; Rendall, Kollias, Ney, & Lloyd, 2005), we attempted to characterise the dominant frequency within sex-specific expected frequency ranges for F4: 3108 and 4250 Hz for males, and 3524-4887 Hz for females, calculated based on published data for male and female formants (Rendall et al., 2005). These data have been used to establish formant thresholds in a previous investigation of the vocal communication of upper-body strength (Puts, Apicella, & Cárdenas, 2012). Minimum values were calculated based on the mean + 0.5 SDs F3 value for (/e/), the vowel with the highest F3 mean and characterised by F3 values 300-700 Hz higher than other vowels. Maximum values were calculated based on the mean + 3 SDs F4 value for (/e/), also the vowel with the highest F4 mean. We chose a liberal maximum as little is known about the resonance properties of nonverbal vocalizations. We consider this measure (hereafter referred to as the dominant formant frequency, DFF4) to be a potential proxy of vocal tract length, as articulatory manipulations of vocal tract shape minimally affect F4 (Rendall et al., 2005), and as the measurement of dominant frequency within an expected F4 range is less likely to capture strong harmonics than for expected ranges of lower formants (as their amplitude declines exponentially with increasing frequency, Titze, 1994). Importantly, F4 is among the strongest formant-based predictors of height in both men and women, explaining a similar amount of variance in height within-sexes as composite formant measures (e.g., formant spacing) and significantly more variance than F1, F2 or F3 (Pisanski, Fraccaro, Tigue, O’Connor, Röder, et al., 2014).

**References**

Abari, K., Rácz, Z. Z., & Olaszy, G. (2011). Formant maps in Hungarian vowels - online data inventory for research, and education. In *Proceedings of Interspeech 2011* (pp. 1609–1612). Florence, Italy. Retrieved from https://www.researchgate.net/profile/Gabor_Olaszy/publication/221484320_Formant_Maps_in_Hungarian_Vowels_-_Online_Data_Inventory_for_Research_and_Education/links/0046351b842a46eb95000000.pdf

Boersma, P., & Weenink, D. (2017). Praat: doing phonetics by computer (Version 5.4). Retrieved from http://www.praat.org/

Fitch, W. T., Neubauer, J., & Herzel, H. (2002). Calls out of chaos: the adaptive significance of nonlinear phenomena in mammalian vocal production. *Animal Behaviour*, *63*(3), 407–418. https://doi.org/10.1006/anbe.2001.1912

Koutseff, A., Reby, D., Martin, O., Levrero, F., Patural, H., & Mathevon, N. (2017). The acoustic space of pain: cries as indicators of distress recovering dynamics in pre-verbal infants. *Bioacoustics*, *0*(0), 1–13. https://doi.org/10.1080/09524622.2017.1344931

Kreiman, J., & Sidtis, D. (2011). *Foundations of voice studies: An interdisciplinary approach to voice production and perception*. Wiley-Blackwell. Retrieved from http://eu.wiley.com/WileyCDA/WileyTitle/productCd-0631222979.html

Paliwal, K. K. (1998). Spectral subband centroid features for speech recognition. In *Proceedings of the 1998 IEEE International Conference on Acoustics, Speech and Signal Processing* (Vol. 2, pp. 617–620). https://doi.org/10.1109/ICASSP.1998.675340

Pisanski, K., Fraccaro, P. J., Tigue, C. C., O’Connor, J. J. M., & Feinberg, D. R. (2014). Return to Oz: Voice pitch facilitates assessments of men’s body size. *Journal of Experimental Psychology: Human Perception and Performance*, *40*(4), 1316–1331. https://doi.org/10.1037/a0036956

Pisanski, K., Fraccaro, P. J., Tigue, C. C., O’Connor, J. J. M., Röder, S., Andrews, P. W., … Feinberg, D. R. (2014). Vocal indicators of body size in men and women: a meta-analysis. *Animal Behaviour*, *95*, 89–99. https://doi.org/10.1016/j.anbehav.2014.06.011

Puts, D. A., Apicella, C. L., & Cárdenas, R. A. (2012). Masculine voices signal men’s threat potential in forager and industrial societies. *Proceedings of the Royal Society of London B: Biological Sciences*, *279*(1728), 601–609. https://doi.org/10.1098/rspb.2011.0829

Reby, D., Levréro, F., Gustafsson, E., & Mathevon, N. (2016). Sex stereotypes influence adults’ perception of babies’ cries. *BMC Psychology*, *4*(1). https://doi.org/10.1186/s40359-016-0123-6

Rendall, D., Kollias, S., Ney, C., & Lloyd, P. (2005). Pitch (F0) and formant profiles of human vowels and vowel-like baboon grunts: the role of vocalizer body size and voice-acoustic allometry. *The Journal of the Acoustical Society of America*, *117*(2), 944–955.

Ryalls, J. H., & Lieberman, P. (1982). Fundamental frequency and vowel perception. *The Journal of the Acoustical Society of America*, *72*(5), 1631–1634. https://doi.org/10.1121/1.388499

Titze, I. R. (1994). *Principles of voice production*. National Center for Voice and Speech.
