# Supplementary material for: Human roars communicate upper-body strength more effectively than do screams or aggressive and distressed speech
Source: PLoS One. 2019 Mar 4;14(3):e0213034. doi: 10.1371/journal.pone.0213034 (PMC6398857; doi:10.1371/journal.pone.0213034)
Supplement: S2 Text — (DOCX) [file pone.0213034.s003.docx]

**S2 Text**

SUPPORTING INFORMATION

for:

Human roars communicate upper-body strength more effectively than do screams or aggressive and distressed speech

Jordan Raine, Katarzyna Pisanski, Rod Bond, Julia Simner, David Reby

**S2 Text: Significant zero-order correlations**

**Significant zero-order correlations between acoustic characteristics and actual strength and height**

***Actual strength***

Female aggressive speech: Major F0 inflections (*r* = .36, *p* = .044), DFF4 (*r* = -.41, *p* = .023)

Female aggressive roar: DFF4 (*r* = -.47, *p* = .007)

Female distressed speech: jitter (*r* = .44, *p* = .012), amplitude modulation (*r* = .50, *p* = .004)

Male distressed speech: time of max intensity (*r* = -.36, *p* = .049)

***Actual height***

Female aggressive speech: Centre of gravity (*r* = .40, *p* = .026)

Female aggressive roar: intensity CV (*r* = .38, *p* = .035)

Female distressed speech: amplitude modulation (*r* = .36, *p* = .049)

Male aggressive speech: F0 CV (*r*  = -.47, *p* = .008)

Male distress scream: DFF4 (*r* = -.40, *p* = .03)

**Significant zero-order correlations between acoustic characteristics and rated strength and height**

***Rated strength***

Female aggressive speech: duration (*r* = .37, *p* = .043), mean F0 (*r* = .67, *p* = < .001), max F0 (*r* = .47,  *p* = .008), min F0 (*r* = .43,  *p* = .015), mean intensity (*r* = .84, *p* < .001), jitter (*r* = .46, *p* = .010), HNR (*r* = -.44, *p* = .013), amplitude modulation (*r* = .72, *p* < .001), centre of gravity (*r =* .40, *p* = .026)

Female aggressive roar: duration (*r* = .41, *p* = .021), inflex (*r* = .36, *p* = .046), mean intensity (*r* = .47, *p* = .007), DFF4 (*r* = -.41, *p* = .024)

Female distressed speech: mean F0 (*r* = .53, *p* = .002), max F0 (*r* = .55, *p*  = .001), minor F0 inflections (*r* = -.41, *p* = .021), mean intensity (*r* = .89, *p* < .001), intensity CV (*r* = .37, *p* = .04), shimmer (*r* = -.41, *p* = .024), amplitude modulation (*r* = .44, *p* = .013), centre of gravity (*r* = .47, *p* = .008)

Female distress scream: mean F0 (*r* = .38, *p* = .035), max F0 (*r* = .36, *p* = .046), min F0 (*r* = .36, *p* = .049), minor F0 inflections (*r* = .46, *p* = .010), mean intensity (*r* = .69, *p* < .001), intensity CV (*r* = -.38, *p* = .034), amplitude modulation (*r* = .43, *p* = .017)

Male aggressive speech: duration (*r* = .41, *p* = .026), mean F0 (*r* = .69, *p* < .001), max F0 (*r*  = .58, *p* = .001), min F0 (*r*  = .58, *p* = .001), F0 CV (*r* = -.51, *p* = .004), mean intensity (*r* = .78, *p* < .001), shimmer (*r* = -.38, *p* = .038), amplitude modulation (*r* = .82, *p* < .001), centre of gravity (*r* = .52, *p* = .003)

Male aggressive roar: mean intensity (*r* = .46, *p* = .010), intensity CV (*r* = -.42, *p* = .022)

Male distressed speech: mean F0 (*r* = .40, *p* = .028), min F0 (*r* = .44, *p* = .014), mean intensity (*r* = .66, *p* < .001), HNR (*r* = -.37, *p* = .044), amplitude modulation (*r* = .69, *p* < .001), centre of gravity (*r*  = .62, *p* < .001)

Male distress scream: mean intensity (*r*  = .60, *p* < .001), intensity CV (*r* = -.46, *p* = .012, HNR (*r* = -.43, *p* = .019), amplitude modulation (*r* = .52, *p* = .003), centre of gravity (*r* = .37, *p* = .044)

***Rated height***

Female aggressive speech: mean intensity (*r* = .49, *p* = .007), HNR (*r* = -.41, *p* - .028), amplitude modulation (*r* = .42, *p* = .025), centre of gravity (*r* = .52, *p* = .004)

Female aggressive roar: mean F0 (*r* = -.49, *p* = .007), max F0 (*r* = -.44, *p* = .018), start-end F0 (*r* = -.38, *p* = .040), F0 CV (*r* = -.42, *p* = .024), mean intensity (*r* = .42, *p*  = .023), centre of gravity (*r* = -.40, *p* = .034)

Female distressed speech: max F0 (*r* = .37, *p* = .041), F0 CV (*r* = .39, *p* = .033), mean intensity (*r* = .63, *p* < .001), intensity CV (*r* = .56, *p* = .001), shimmer (*r* = -.48, *p* = .006), jitter (*r* = .41, *p* = .023), HNR (*r* = -.39, *p* = .030), amplitude modulation (*r* = .52, *p* = .003)

Female distress scream: mean F0 (*r* = -.41, *p* = .02), min F0 (*r* = -.38, *p* = .036), centre of gravity (*r* = -.47, *p* = .008)

Male aggressive speech: mean intensity (*r* = .38, *p* = .04)

Male aggressive roar: mean F0 (*r* = -.53, *p* = .004), max F0 (*r* -.49, *p* = .008), time of max amplitude (*r* = -.40, *p* = .036), jitter (*r* = .43, *p* = .023),

Male distressed speech:

Male distress scream: mean F0 (*r*  = -.58, *p* = .001), max F0 (*r* = -.57, *p* = .001), min F0 (*r* = -.46, *p* = .011)
